# The Cysteine Rich Necrotrophic Effector SnTox1 Produced by *Stagonospora nodorum* Triggers Susceptibility of Wheat Lines Harboring *Snn1*

**DOI:** 10.1371/journal.ppat.1002467

**Published:** 2012-01-05

**Authors:** Zhaohui Liu, Zengcui Zhang, Justin D. Faris, Richard P. Oliver, Robert Syme, Megan C. McDonald, Bruce A. McDonald, Peter S. Solomon, Shunwen Lu, Weilin L. Shelver, Steven Xu, Timothy L. Friesen

**Affiliations:** 1 Department of Plant Pathology, North Dakota State University, Fargo, North Dakota, United States of America; 2 Northern Crop Science Laboratory, USDA-ARS, Fargo, North Dakota, United States of America; 3 Department of Environment & Agriculture, Curtin University, Perth, Australia; 4 Plant Pathology Group, Institute of Integrative Biology, Swiss Federal Institute of Technology (ETH), Zurich, Switzerland; 5 Research School of Biology, The Australian National University, Canberra, Australia; 6 Biosciences Research Laboratory, USDA-ARS, Fargo, North Dakota, United States of America; Virginia Polytechnic Institute and State University, United States of America

## Abstract

The wheat pathogen *Stagonospora nodorum* produces multiple necrotrophic effectors (also called host-selective toxins) that promote disease by interacting with corresponding host sensitivity gene products. SnTox1 was the first necrotrophic effector identified in *S. nodorum*, and was shown to induce necrosis on wheat lines carrying *Snn1*. Here, we report the molecular cloning and validation of *SnTox1* as well as the preliminary characterization of the mechanism underlying the SnTox1-*Snn1* interaction which leads to susceptibility. *SnTox1* was identified using bioinformatics tools and verified by heterologous expression in *Pichia pastoris*. *SnTox1* encodes a 117 amino acid protein with the first 17 amino acids predicted as a signal peptide, and strikingly, the mature protein contains 16 cysteine residues, a common feature for some avirulence effectors. The transformation of *SnTox1* into an avirulent *S. nodorum* isolate was sufficient to make the strain pathogenic. Additionally, the deletion of *SnTox1* in virulent isolates rendered the *SnTox1* mutated strains avirulent on the *Snn1* differential wheat line. *SnTox1* was present in 85% of a global collection of *S*. *nodorum* isolates. We identified a total of 11 protein isoforms and found evidence for strong diversifying selection operating on *SnTox1*. The SnTox1-*Snn1* interaction results in an oxidative burst, DNA laddering, and pathogenesis related (PR) gene expression, all hallmarks of a defense response. In the absence of light, the development of SnTox1-induced necrosis and disease symptoms were completely blocked. By comparing the infection processes of a GFP-tagged avirulent isolate and the same isolate transformed with *SnTox1*, we conclude that SnTox1 may play a critical role during fungal penetration. This research further demonstrates that necrotrophic fungal pathogens utilize small effector proteins to exploit plant resistance pathways for their colonization, which provides important insights into the molecular basis of the wheat-*S. nodorum* interaction, an emerging model for necrotrophic pathosystems.

## Author Summary

In this manuscript we describe the cloning of *SnTox1* from *Stagonospora nodorum*, the gene encoding the first host selective toxin (SnTox1) identified in this fungus. SnTox1 induces necrosis and promotes disease on wheat lines harboring the *Snn1* gene. We verified the function of the SnTox1 gene by expressing it in a yeast culture where the resulting culture filtrate induced necrosis but only on wheat lines that carried a functional *Snn1*. The *SnTox1* gene was also transformed into an avirulent *S. nodorum* isolate, resulting in an isolate that was virulent on wheat lines harboring *Snn1*. *SnTox1* was also disrupted in virulent *S. nodorum* isolates resulting in the elimination of disease on *Snn1* differential wheat lines. Additionally, we investigated the host response to SnTox1 and *S. nodorum* strains producing SnTox1 and discovered that several hallmarks of a resistance response were present during the susceptible reaction, showing that the necrotrophic pathogen *S. nodorum* is likely using SnTox1 to stimulate a host resistance pathway involving *Snn1* to induce disease.

## Introduction

Like other parasites, fungal pathogens secrete a battery of molecules known as effectors during the infection process. These effectors can alter plant biological processes resulting in successful colonization [1], [2]. Conversely, recognition of effectors by the plant innate immune system can initiate a defense response resulting in effector-triggered immunity (ETI) [3], [4]. ETI is characterized by the accumulation of reactive oxygen species (ROS), transcriptional induction of pathogenesis-related (PR) genes and production of antimicrobial chemical compounds, eventually leading to rapid and localized plant cell death, known as the hypersensitive response (HR) [5]. In ETI, the perception of the fungal effector is mediated by the corresponding plant resistance gene (R) which acts in a gene-for-gene manner [6], [7]. Currently, it is believed that this localized suicide of plant cells induced by ETI halts further growth of the biotrophic fungal pathogen, which requires living plant tissue for survival.

Necrotrophic fungal pathogens are known to produce host selective toxins (HSTs), including low molecular weight metabolites and small secreted proteins that function as essential determinants of pathogenicity or virulence [8], [9]. HSTs can therefore be viewed as effectors of necrotrophic pathogenicity and hence we prefer the term necrotrophic effector (NE) [10], [11]. These effectors play significant roles in determining the outcomes of plant-pathogen interactions by specifically interacting (directly or indirectly) with the products of corresponding host genes [12], [13]. However, in contrast to ETI in the classical gene-for-gene model, the necrosis induced by effectors from necrotrophic fungal pathogens results in disease susceptibility; thus, it can be described as effector-triggered susceptibility (ETS) [14], [15], a term which was originally used in reference to biotrophic systems [4].

The molecular basis of necrosis-induced ETS involving necrotrophic fungi is still largely unknown, but has in several cases exhibited the hallmarks of programmed cell death (PCD); DNA laddering, heterochromatin condensation, cell shrinkage, callose deposition and an oxidative burst [9], [16], [17]. ToxA, a necrotrophic effector found in both *Pyrenophora tritici-repentis* and *Stagonospora nodorum,* causes the loss of plasma membrane integrity and the accumulation of hydrogen peroxide [18], [19]. Microarray analysis revealed that several wheat genes involved in defense response and signaling pathways were strongly regulated by the ToxA-*Tsn1* interaction [20], [21].

Interestingly, three plant genes involved in susceptibility to necrotrophic effectors (*Pc*, the sorghum sensitivity gene corresponding to PC toxin; *LOV1*, the *Arabidopsis* sensitivity gene corresponding to victorin; and *Tsn1*, the wheat sensitivity gene corresponding to ToxA) have been cloned and shown to be resistance-like genes containing both nucleotide binding (NB) and leucine-rich repeat (LRR) domains [15], [22], [23]. This has led to speculation that necrotrophic fungal pathogens may utilize plant resistance signaling pathways to subvert PCD and enable pathogen growth [15], [24].

*Stagonospora nodorum*, an ascomycete fungus (teleomorph: *Phaeosphaeria nodorum*), is the causal agent of wheat Stagonospora nodorum blotch (SNB), a globally distributed and economically important disease [25]. *S. nodorum* is a typical necrotrophic fungal pathogen [10], [26]. In recent years, it has been shown that this pathosystem is based largely on interactions involving proteinaceous necrotrophic effectors and corresponding host sensitivity genes that, when occurring together, result in ETS. To date, six interactions have been reported including SnTox1-*Snn1* [27], SnToxA-*Tsn1* [28], [29], SnTox2-*Snn2* [12], SnTox3-*Snn3-B1* [30], SnTox4-*Snn4* [31], and SnTox3-*Snn3-D1* [32]. In addition, several other effector-host gene interactions have been identified (Friesen and Faris, Oliver and Tan, unpublished data). Therefore, the wheat-*S*. *nodorum* system is emerging as a model to investigate the molecular mechanisms of necrotrophic pathogenesis [13]. One of our research goals has been to clone necrotrophic effector genes and decipher their molecular and biochemical functions.

Of the *S. nodorum* effector genes, *SnToxA* and *SnTox3* have been cloned with the aid of the *S. nodorum* genome sequence information [14], [29], [33]. The *SnToxA* gene is essentially identical to the *ToxA* gene isolated from the wheat tan spot pathogen *P. tritici-repentis.* Mature ToxA consists of a 13.2 kDa protein containing two cysteine residues as well as an RGD-containing vitronectin-like motif that is present in a solvent-exposed loop in the active protein [34]–[38]. The RGD motif has been shown to be essential for internalization and internalization has been shown to be critical for the induction of necrosis [37], [39], [40] *SnTox3* encodes an approximately 17.5 kDa mature protein with six cysteine residues and has no homology to genes in the public databases [14].

Here, we report the molecular cloning and characterization of the *SnTox1* gene which encodes the SnTox1 protein, and we show that SnTox1 is specifically recognized by the corresponding wheat sensitivity/susceptibility gene *Snn1*. The characterization of the SnTox1-*Snn1* interaction provides strong evidence that necrotrophic fungal pathogens use necrotrophic effectors to subvert the host resistance mechanism to cause disease.

## Results

### *SNOG_20078* was identified as the SnTox1-encoding gene

Whole genome reference sequences have proven to be powerful for the identification of fungal and oomycete effector genes [1], [41]. The annotated *S. nodorum* genome sequence supports a minimum of 10,762 nuclear genes with 1,782 predicted to encode extracellular proteins [33]. A specific set of criteria was used to prioritize the genes and generate a list of candidates. The criteria (size less than 30 kDa, predicted to be secreted, expressed in planta, etc, see Materials and Methods) were based on the characteristics of the previously cloned *SnToxA* and *SnTox3* genes. We focused on the top 100 genes and as expected, *SnTox3* and *SnToxA* were identified among them. PCR analysis was conducted to confirm the absence of genes in the *S*. *nodorum* avirulent isolate Sn79-1087 (data not shown). Genes meeting these criteria were expressed in the *Pichia pastoris* heterologous expression system [14]. This process and the subsequent screening of a set of differential lines (see Materials and Methods) led us to identify *SNOG_20078* as the SnTox1-encoding gene.

**Figure 1:**
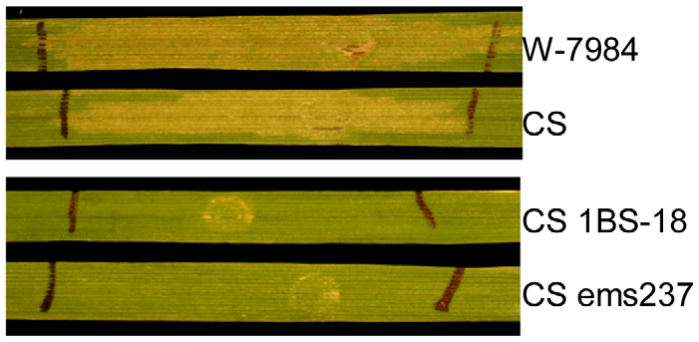
*SNOG_20078* encodes SnTox1. The reaction of wheat lines W-7984 (*Snn1*), Chinese Spring (CS) (*Snn1*), CS 1BS-18 (*snn1*) and CS ems237 (*snn1*) to culture filtrates of a *Pichia pastoris* strain transformed with *SNOG_20078*.

Culture filtrates of *P*. *pastoris* strain X33 transformed with the coding region of *SNOG_20078* cDNA were infiltrated into the leaves of W-7984, Chinese Spring (CS), CS 1BS-18 and CS ems237. W-7984 and CS carry the dominant *Snn1* allele that confers sensitivity to SnTox1 [27]. CS 1BS-18 and CS ems237 are nearly identical to CS, but harbor mutations at the *Snn1* locus, resulting in insensitivity to SnTox1. Necrosis developed in the SnTox1-sensitive lines W-7984 and CS, but not in CS 1BS-18 and CS ems237 (Figure 1) suggesting that *SNOG_20078* was the SnTox1-encoding gene. To map the gene conferring sensitivity, the same culture filtrates were subsequently infiltrated into the entire ITMI mapping population, which segregates for *Snn1*/*snn1*. All lines sensitive to the partially purified native SnTox1 [27] were also sensitive to the culture filtrates of the *SNOG_20078* transformed yeast strain. This strongly indicated that *SNOG_20078* was the SnTox1-encoding gene and therefore we designated it *SnTox1*.

### *SnTox1* gene structure and genomic location

**Figure 2:**
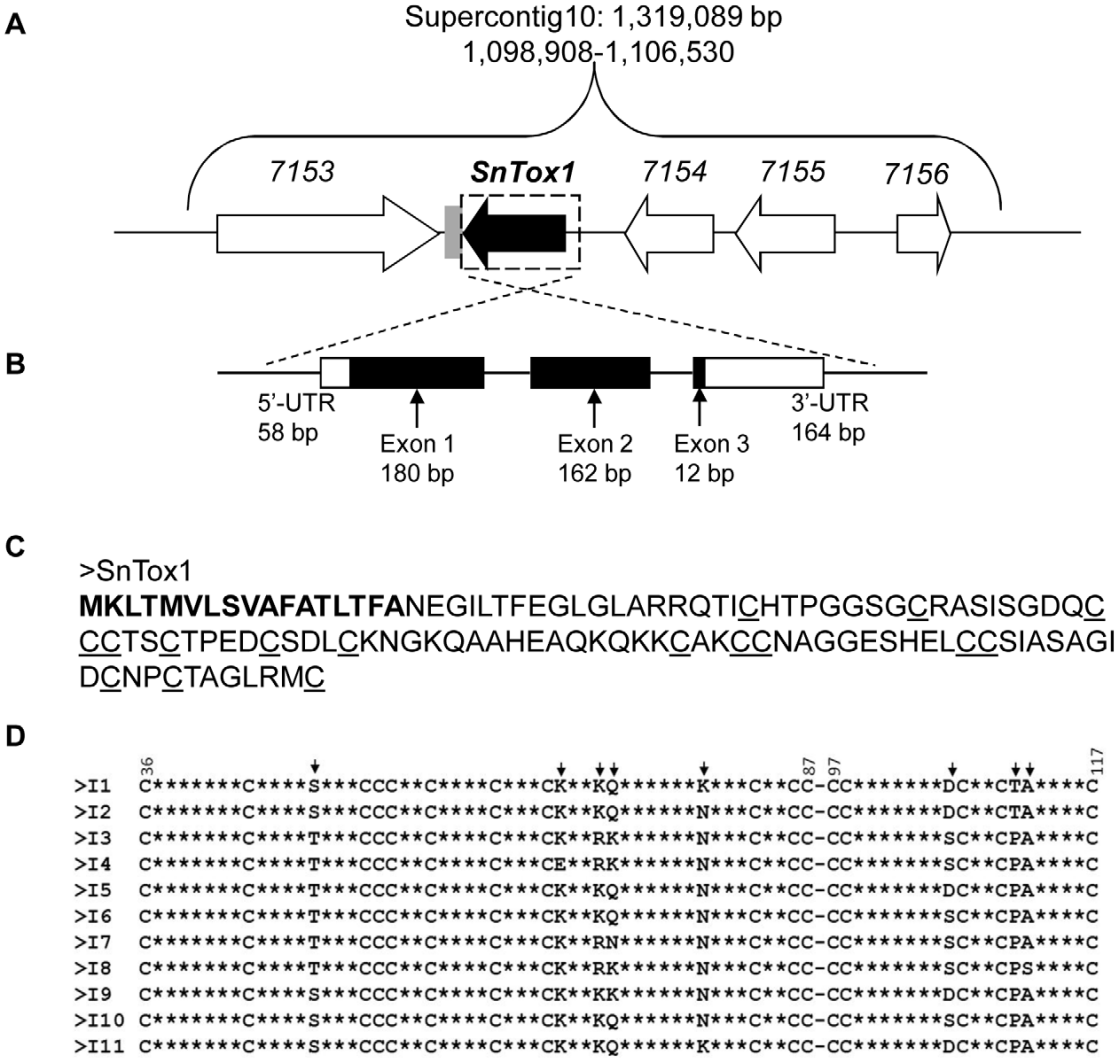
Genomic location, structure and deduced amino acid sequence of *SnTox1*. **A**. *SnTox1* genomic region. *SnTox1* is located in supercontig 10 of the assembled SN15 genome sequence and is surrounded by four other predicted genes (boxed arrows, SNOG7153 to SNOG7156). A short truncated molly-type retrotransposon sequence (gray rectangle) closely follows *SnTox1*. **B**. *SnTox1* gene structure. The full length transcript of *SnTox1* and contains three exons (black rectangles) and both 5′ and 3′ untranslated regions (white rectangles). **C**. SnTox1 amino acid sequence. SnTox1 protein contains 117 amino acids with the first 17 (in bold) being a predicted signal sequence. The 16 cysteine residues are underlined. **D**. The alignment of 11 different SnTox1 protein isoforms. Two regions of SnTox1, from 36 to 87 and 97 to 117 are shown to indicate the variable amino acid positions (arrows). The remaining amino acids, (except for the cysteine residues) are shown as stars, indicating they are conserved.

*SnTox1* is located in supercontig 10 of the assembled SN15 genome sequence ([33], Figure 2A). Within a ≈7.6 kb region, there are three genes upstream (*SNOG_07154*-*SNOG_7156*) and one downstream (*SNOG_07153*) of *SnTox1* (Figure 2A). Similarly, there is a short truncated molly-type retrotransposable element (183 bp) sequence following *SnTox1* (http://genome.jgi-psf.org/cgi-bin/browserLoad/?db=Stano1&position=scaffold_10). The sequencing of the 5′ and 3′ RACE fragments revealed three exons as well as 5′ and 3′ untranslated regions (UTRs) in the full-length transcript of *SnTox1* (Figure 2B). Putative TATA and CAAT boxes were identified 114 bp and 570 bp upstream of the ATG start site, respectively (Figure S1).

The SnTox1 protein consists of 117 amino acids with the first 17 amino acids predicted as a signal peptide. Interestingly, 16 of the remaining 100 amino acids are cysteine residues (Figure 2C). No prosequence was predicted using the web-based program ProP 1.0 (http://www.cbs.dtu.dk/services/ProP/) and after the cleavage of the predicted signal sequence the mature protein was estimated to be 10.33 kDa. To demonstrate that SnTox1 was produced in yeast culture and to verify the size of SnTox1, we applied western blot analysis to the protein samples prepared from SnTox1 yeast culture filtrates. The antibody for SnTox1 was generated from rabbit immunized with a BSA-conjugated 14 amino acid long SnTox1 peptide (see Material and Methods). A single band was only observed in protein samples prepared from SnTox1 yeast culture filtrates, but not from the control culture filtrates (yeast strain transformed with an empty vector). Furthermore, the western band was visualized between the size standard of 10 and 15 kDa, but much closer to 10 kDa (Figure S2). The estimated size of SnTox1 obtained from the western blot agreed with the predicted molecular weight of 10.33 kDa for the mature protein.

A BlastP search of the NCBI non-redundant database with the SnTox1 protein sequence as a query led to the identification of three putative proteins with unknown functions, one from *S*. *nodorum* (SNOG_06487) and two from *P*. *tritici-repentis* (PTRT_04748 and PTRT_03544) with similarities of 38%, 56%, and 43%, respectively. The conserved amino acids between SnTox1 and these proteins were mostly distributed in the predicted signal sequence and the N-terminal region of the mature protein (Figure S3).

### SnTox1 contains a C-terminal chitin-binding-like motif

**Figure 3:**
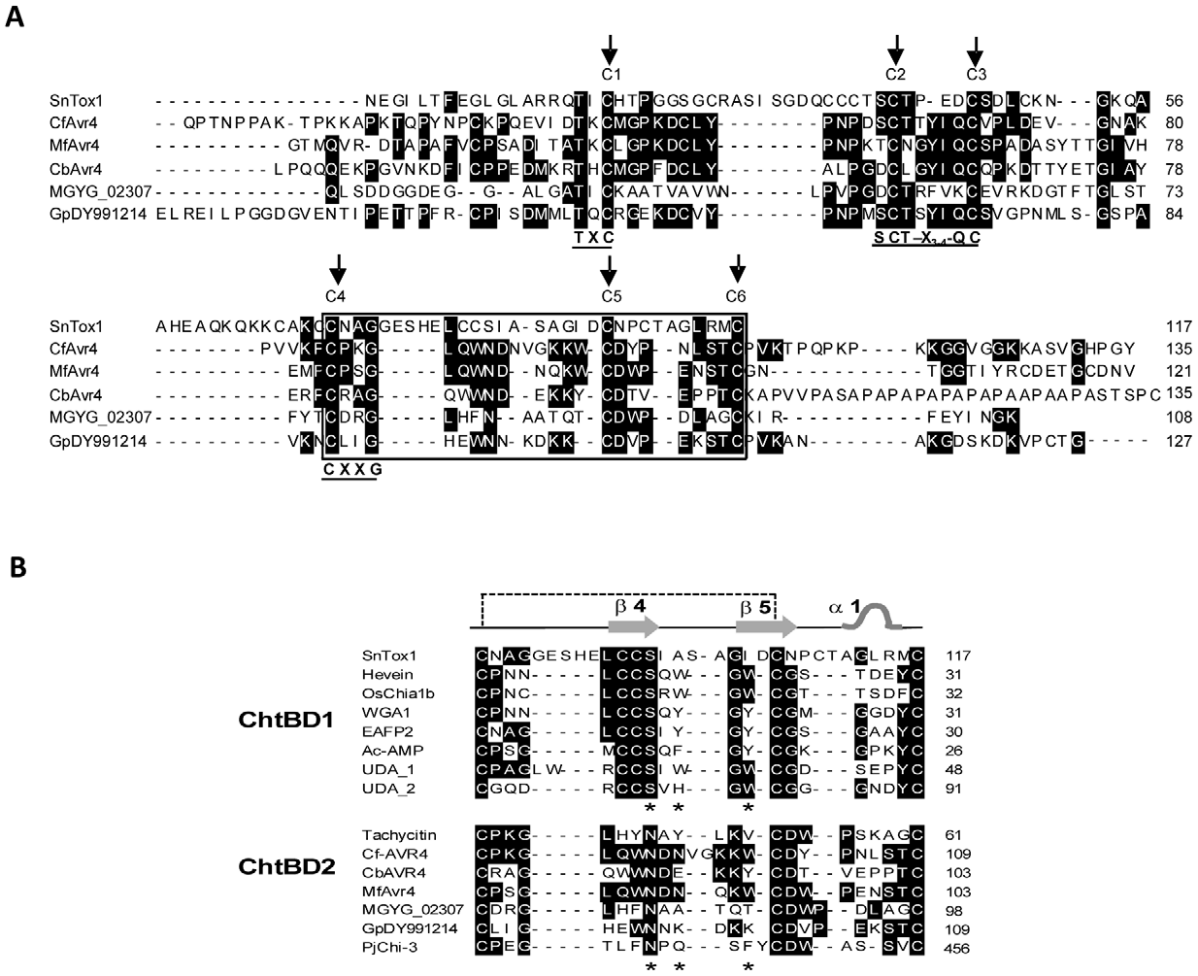
Sequence alignment of SnTox1 with other proteins harboring chitin binding domains. **A**. Sequence alignment of SnTox1 with Avr4-like proteins. Three motif-like sequences (TxC at C1, SCT-x-QC at C2 and C3, and CxxG at C4) and six conserved cysteine residues are marked by arrows. CfAvr4 =  *Cladosporium fulvum* Avr4 protein (CAA69643.1), MfAvr4 =  *Mycosphaerella fijiensis* Avr4-like protein (Protein ID: Mycfi1:87167), CbAvr4 = *Cercospora beticola* Avr4-like protein (GU574324), MGYG_02307 =  Avr4-like protein from *Microsporum gypseum* and GpDY991214 =  Avr4-like protein identified from *Geomyces pannorum*. **B**. Local alignment of chitin binding (CB) domains from different proteins. Positions of the two antiparallel β-sheets (β4 and β5, arrows), helical turn (α1), disulfide bond (dashed line), and the active sites (asterisks) conserved in CB domains (based on Suetake et al., [44]) are indicated at the top and/or the bottom, respectively. SnTox1 contained all secondary-structure-related residues including the strictly conserved β-strand-forming “CCS” motif found only in plant-specific ChtBD1 proteins.

Amino acid alignment with manual adjustment indicated that SnTox1 contained local similarity with cysteine-rich *Cladosporium fulvum* Avr4-like fungal effectors (Figure 3A) from *Cercospora beticola*, *Mycosphaerella fijiensis* [42] and two ascomycete human pathogens, *Microsporum gypseum* and *Geomyces pannorum* (this study). These conserved motifs were identified within the chitin-binding domain (ChtBD) including the C-terminal conserved chitin-binding (CB) motif (Figure 3A). Three-dimensional (3D) structure-based sequence alignment suggested that the putative CB motif in SnTox1 was more similar to those of plant-specific ChtBDs (ChtBD1, or CBM18 superfamily, pfam00187) than to Avr4 proteins, which are related to invertebrate ChtBDs (ChBD2, or the CBM14 superfamily, pfam01607) [43] (Figure 3B). SnTox1 contained all secondary-structure-related residues including the strictly conserved β-strand-forming “CCS” motif found only in plant-specific ChtBD1 proteins [44] (Figure 3B). In contrast, all Avr4-like proteins lacked the “CCS” motif and had a loosely conserved “QWN” motif at the same positions as that found in the antimicrobial protein tachycitin, a representative ChtBD2 [44]. There were several insertions found between conserved regions in SnTox1 which also lacked the C-terminal extension after the conserved CB motif, suggesting a significant sequence divergence between SnTox1 and Avr4-like proteins.

### *SnTox1* is present in most virulent isolates and absent in avirulent isolates

**Figure 4:**
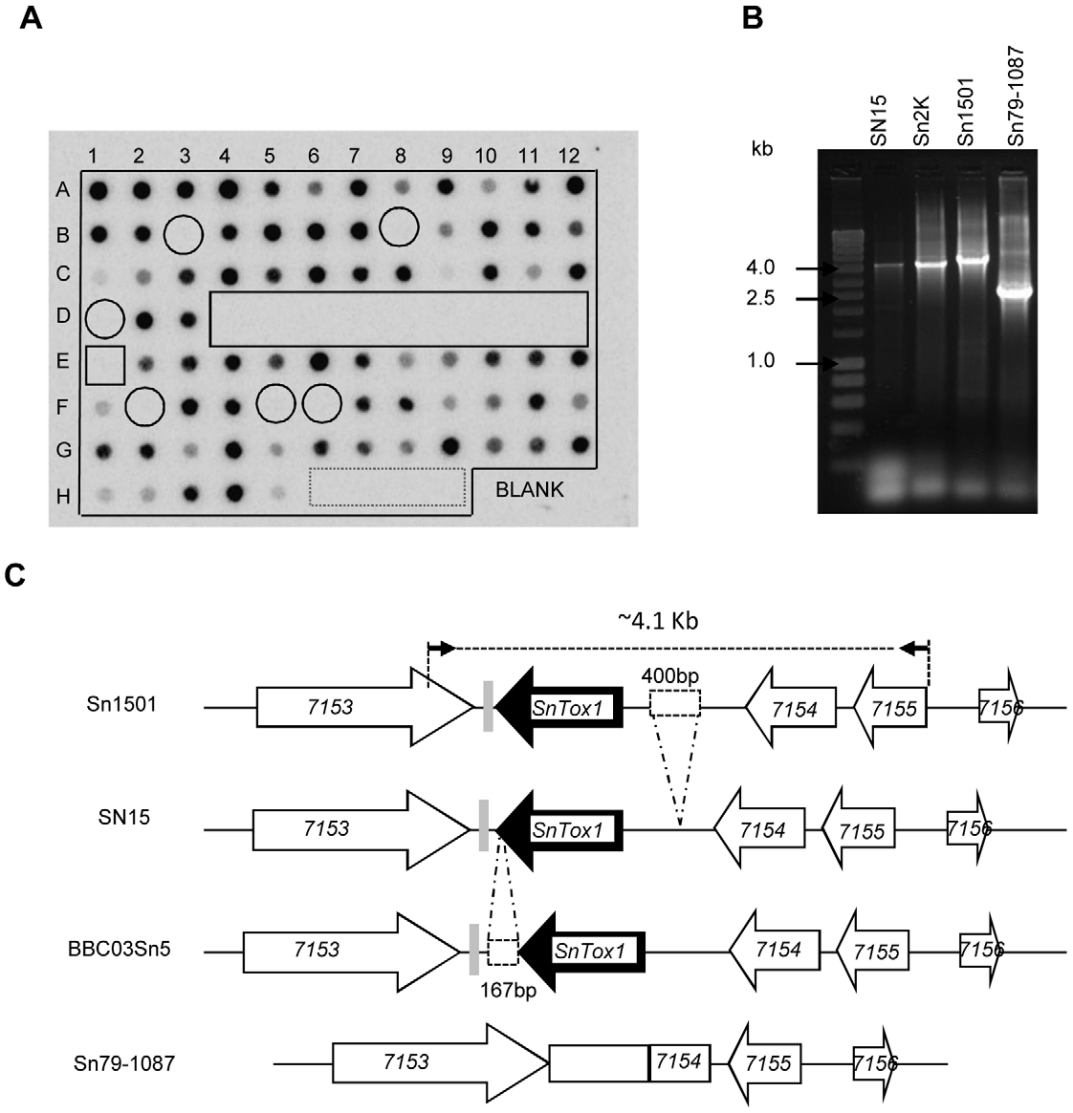
Distribution of *SnTox1* in *S. nodorum* isolates and variation in its genomic region. **A**. DNA dot blot analysis of *SnTox1* gene distribution. Among 93 fungal isolates or species (Table S1), *SnTox1* is absent in related fungal species (dash-lined box), *S*. *nodorum* avirulent isolates (box), and 6 virulent isolates not containing *SnTox1*(circles). The remaining virulent isolates (72 out of 79) all contained *SnTox1*. **B**. PCR amplification of the ∼4.1 kb genomic region of *SnTox1* in different *S*. *nodorum* isolates. Two primers located within *SNOG_7153* and *SNOG_7155* (see Fig 4C arrows for primer locations) were used to amplify the *SnTox1* genomic region in four *S. nodorum* isolates including the avirulent isolate (Sn79-1087). A difference in fragment size was observed among isolates. **C**. A diagram of the *SnTox1* genomic region in different *S. nodorum* isolates. Five predicted genes (arrow blocks) and a truncated molly type repeat (gray rectangle) are schematically drawn. The 4.1 Kb region was amplified with two primers in *SNOG_7153* and *SNOG_7155* (small black arrows) and was investigated by cloning and sequencing. In Sn79-1087, a portion of *SNOG_7154* and the entire *SnTox1* gene was missing and was replaced by a 1.3 kb region (rectangle). Additionally, two indels (dash-lined rectangle) were identified in the upstream region and the 3′UTR of *SnTox1*.

The distribution of *SnTox1* in different *S*. *nodorum* isolates and related fungal species (Table S1 and S2) was investigated using PCR assays and DNA dot blots. Among the 777 isolates that were sampled from wheat fields around the world, 85% (661) possess the *SnTox1* gene (Table S1). Dot blot analysis of a subset of a global collection (Table S2) showed that *SnTox1* was absent in all *S. nodorum* isolates collected from wild grasses which are avirulent on wheat (Figure 4A). Additionally, *SnTox1* was absent in related fungal species including *P. tritici-repentis*, *P*. *teres*, *P*. *bromi* and *M. graminicola*.

To investigate sequence variation in *SnTox1*, the gene was PCR-amplified and sequenced from 159 global *S. nodorum* isolates. We found 12 different nucleotide haplotypes, 11 of which encode different protein isoforms, consistent with strong diversifying selection. The 11 protein isoforms involve amino acid changes at eight positions within SnTox1; however, all cysteine residues remain unchanged across all isoforms (Figure 2D). The nucleotide sequences of all 12 haplotypes have been submitted to GenBank and the accession number for each haplotype is provided at the end of the text. Four codons exhibit significant positive selection using PAML (Table 1). These findings provide strong evidence that positive diversifying selection, consistent with a co-evolutionary process, has been operating on *SnTox1*.

**Table 1:** Summary of a likelihood ratio test using PAML for positive selection in the *SnTox1* gene.

Model pairs	*d_N_*/*d_S_*	Log likelihood (*l*)^a^	*P* b	Positively selected codons c	Posterior prob.
M1a (nearlyneutral)	0.52	−553.98		48	0.975
M2a (selection)	4.49	−546.04**		68	0.978
M1a/M2a LRT	**<0.001**			72	0.997
M7 (beta)	0.50	−554.07		106	0.974
M8 (beta & ω)	4.49	−546.127**			
M7/M8 LRT	**<0.001**				

a Asterisks indicate which model resulted in a statistically higher likelihood score for the *SnTox1* codon alignment.

b P-value for the likelihood ratio test (LRT) between the log-likelihoods of comparable models. In both cases the null hypothesis is rejected in favor of the selection model.

c Specific codons within the amino acid sequence were tested for positive selection. Positively selected codons with a Bayes Empirical Bayes posterior probability higher than 0.95 are listed on the right half of the table [78].

To investigate sequence variation of the *SnTox1* genomic region in virulent and avirulent isolates, we used PCR to amplify the four genes flanking *SnTox1* (*SNOG_07153*, *SNOG_07154*, *SNOG_07155*, and *SNOG_07156*, see Figure 2A for their locations). Only *SNOG_07154* located directly upstream of *SnTox1* could not be amplified from the avirulent isolate Sn79-1087 (data not shown), which suggested that a region containing all or part of *SNOG_07154* as well as the entire *SnTox1* sequence may be missing in Sn79-1087. PCR primers were designed within the two genes *SNOG_07153* and *SNOG_07155* and used to amplify DNA from different virulent isolates as well as Sn79-1087. The amplified fragment in SN15 was ∼4.1 kb as expected but 4.5 kb in Sn1501 and 2.3 kb in Sn79-1087 (Figure 4B). Sequencing revealed that a 3.1 kb region including *SnTox1* and the last 85 bp of the 3′ end of *SNOG_07154* coding region was replaced by a 1.3 kb sequence in Sn79-1087 (Figure 4C). The 1.3 kb insertion in Sn79-1087 does not share homology with any other known sequence in the NCBI database. Sequence analysis also revealed that two indels occur in the *SnTox1* genomic region with one indel of 400 bp in the upstream, and the other indel of 167 bp at the end of the 3′UTR region (Figure 4C).

### Addition of the *SnTox1* gene to an avirulent isolate is sufficient to change the host range

**Figure 5:**
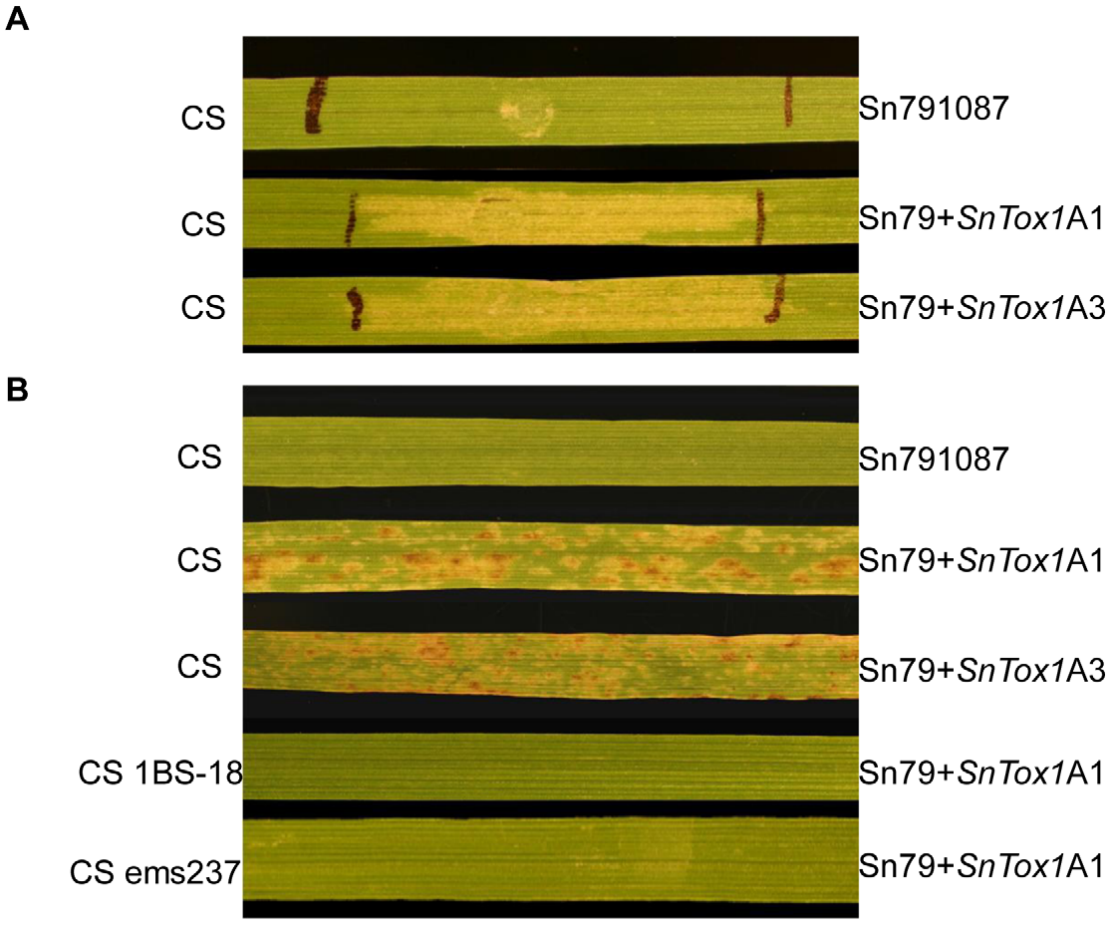
*SnTox1* makes an avirulent isolate pathogenic. **A**. Reaction of Chinese Spring to infiltration with culture filtrates from the avirulent isolate Sn79-1087 and its *SnTox1* transformants. The culture filtrates from Sn79-1087 did not produce necrosis on CS, but the two *SnTox1* transformants Sn79+SnTox1A1 and Sn79+SnTox1A3 did. **B**. Disease reaction of CS (*Snn1*) and CS 1BS-18 (*Snn1* deleted) or CS ems237 (*Snn1* mutated) to the inoculation with the avirulent isolate Sn79-1087 and its *SnTox1* transformants. Sn79-1087 did not cause disease on CS, but the two Sn79+SnTox1A1 and Sn79+SnTox1A3 did produce tan necrotic lesions and widespread flecking on CS (*Snn1*). However, the two transformants were unable to cause disease on CS 1BS-18 and CS ems237 which carry the recessive allele (*snn1*).

The avirulent isolate Sn79-1087 does not produce any known *S*. *nodorum* necrotrophic effectors, nor does it induce a susceptible response on any of the wheat lines that we have tested. Therefore, a 1.1 kb *SnTox1* genomic region (Figure S1) containing the native promoter, open reading frame, and the native terminator was cloned into the pDAN vector (Figure S4A) and transformed into Sn79-1087. Southern blot analysis indicated all but one transformant possessed the 1.1 kb *SnTox1* fragment (Figure S4B). Transformants A1 and A3, designated *Sn79+SnTox1A1* and *Sn79+SnTox1A3*, were selected for further analysis. We confirmed that culture filtrates of Sn79-1087 did not cause necrosis nor did spore inoculations cause disease on CS, which contains *Snn1* (Figure 5A). However, infiltration of culture filtrates from *Sn79+SnTox1A1* and *Sn79+SnTox1A3* produced necrosis on the leaves of CS (Figure 5A) and inoculation of CS with conidia of *Sn79+SnTox1A1* and *Sn79+SnTox1A3* produced lesions on the leaves of CS (Figure 5B). The two transformants did not cause disease on CS 1BS-18 or CS ems237, which lack a functional *Snn1* gene (Figure 5B).

### Deletion of *SnTox1* in virulent isolates renders them nonpathogenic on *Snn1* differential lines

The virulent isolate Sn2000 was used in the original identification of SnTox1 and *Snn1* [27]. Therefore, this isolate was used to conduct gene knock outs of *SnTox1*. We exploited a PCR-based split marker method to replace the majority of the *SnTox1* gene with the hygromycin resistance gene (*hyg^R^*). The transformants were verified using Southern blot analysis with a probe amplified from the *SnTox1* region that was replaced by *hyg^R^* (Figure S4C). In two transformants designated *Sn2000ΔSnTox1*–*9* and *Sn2000ΔSnTox1*–*15*, the *SnTox1* gene was successfully replaced, and one transformant designated *Sn2000ΔSnTox1-ECT* was identified as an ectopic insertion due to it being hygromycin resistant but still having an intact and functional *SnTox1* gene (Figure S4D).

**Figure 6:**
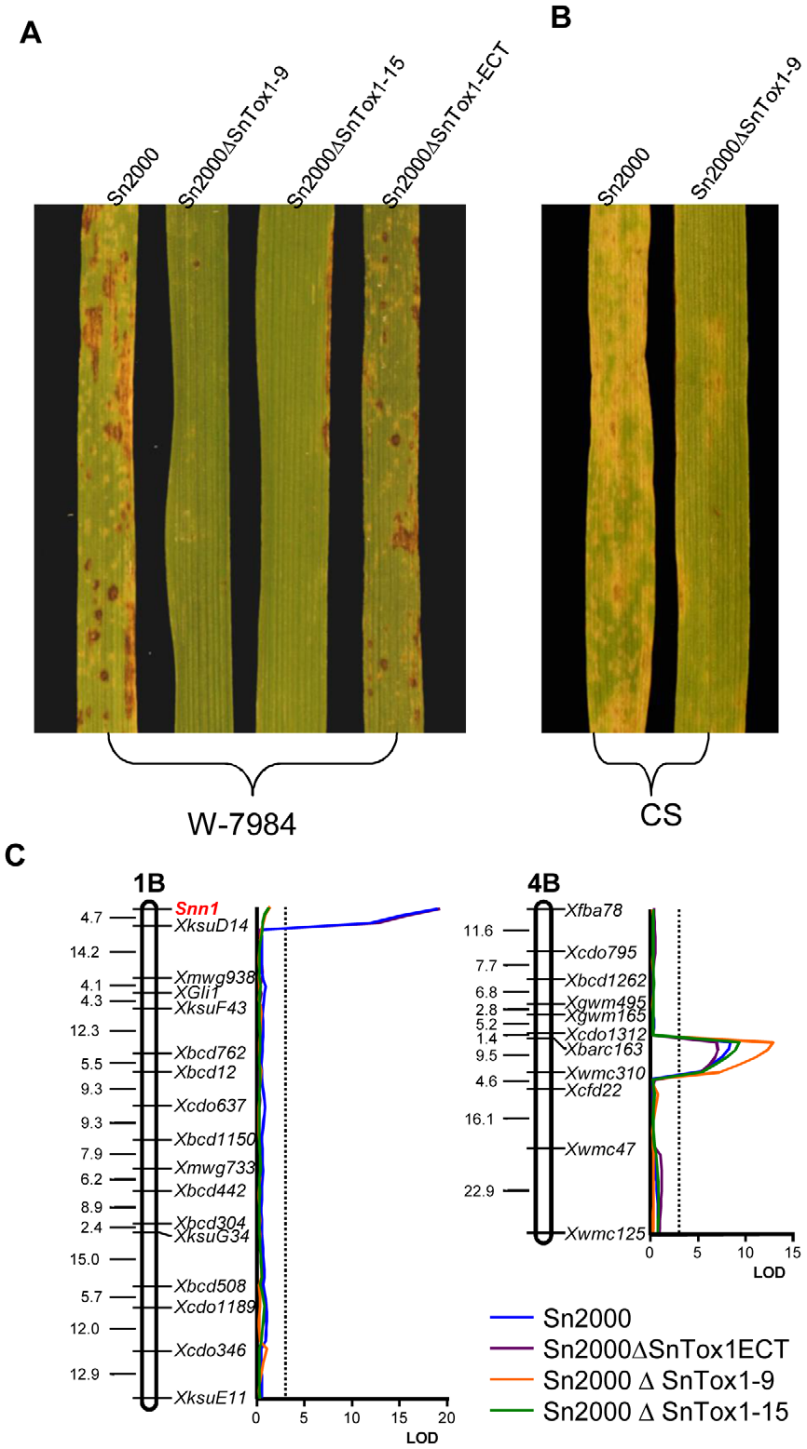
*SnTox1* disruption affects virulence only on *Snn1* differential lines. **A**. Reaction of W-7984 to inoculation with Sn2000 and its *SnTox1* disrupted (Sn2000*Δ*SnTox1-9 and Sn2000*Δ*SnTox1-15) and ectopic (Sn2000*Δ*SnTox1-ECT) strains. Compared to the wild type and ectopic strain, the two *SnTox1* disrupted strains completely lost virulence on W-7984 which only contains the SnTox1 sensitivity gene (*Snn1*). **B**. Reaction of CS to inoculation with Sn2000 and its *SnTox1* disrupted strain (Sn2000*Δ*SnTox1-9). Compared to wild type, the *SnTox1* disrupted strain (Sn2000*Δ*SnTox1-9) showed significantly reduced virulence on CS which is not only sensitive to SnTox1 but to another necrotrophic effector produced by Sn2000. **C**. Interval map of chromosome 1B (left) and 4B (right) from QTL mapping in the ITMI population inoculated with Sn2000, and its *SnTox1* disrupted and ectopic strains. Strains are depicted by different colors as indicated. A centiMorgan scale is on the left of the map and markers are shown in their relative position along the right. The *Snn1* locus on the tip of chromosome 1B is shown in red. An LOD scale is shown along the x axis, and the critical LOD threshold of 3.0 is represented by the dotted lines.

Spores of the three transformed fungal strains along with wild type Sn2000 were inoculated onto the *Snn1* differential wheat line W-7984 [27]. The ectopic strain *Sn2000ΔSnTox1-ECT* induced similar reaction as the wild type including defined tan necrotic lesions with widespread small white flecking, whereas the two knockout strains induced almost no reaction on the leaves (Figure 6A) indicating SnTox1 is an important virulence factor for Sn2000. *Sn2000ΔSnTox1*–*9* and the Sn2000 wild type were also inoculated onto CS. Compared to the wild type, the virulence of *Sn2000ΔSnTox1*–*9* on CS was substantially reduced, but not completely eliminated (Figure 6B), which is due to CS having at least one additional necrotrophic effector sensitivity gene that likely interacts with another effector produced by Sn2000 (Faris and Friesen, unpublished).

The wheat ITMI population was used to originally map the QTL associated with disease susceptibility caused by Sn2000, in which two significant QTL were identified, one on chromosome 1BS corresponding to the *Snn1* locus and the other on chromosome 4BL, explaining 48% and 9% of the disease, respectively [45]. We inoculated the three fungal strains: *Sn2000ΔSnTox1*–*9*, *Sn2000ΔSnTox1*–*15* and *Sn2000ΔSnTox1-ECT* along with wild type Sn2000 onto the ITMI population. For Sn2000, as expected, we detected two significant QTL with one being at the *Snn1* locus and the other being on chromosome 4BL accounting for 50 and 17% of the disease variation, respectively. A very similar result was obtained for *Sn2000ΔSnTox1-ECT* where the *Snn1* QTL and the QTL on chromosome 4BL were detected explaining 50 and 15% of the variation, respectively (Figure 6C). However, in the inoculation with the two *SnTox1* knock out strains, the QTL conferred by *Snn1* on chromosome 1BS became undetectable showing no association with disease, but the QTL on chromosome 4B was retained and became more significant overall accounting for 40–50% of the disease variation (Figure 6C). The QTL analysis in the ITMI population clearly demonstrated that *SnTox1* codes for the SnTox1 protein which plays a significant role in disease by interacting with the host gene *Snn1*.

### The transcription of *SnTox1* peaks at 3 days post inoculation correlating with the onset of necrotic lesion development

**Figure 7:**
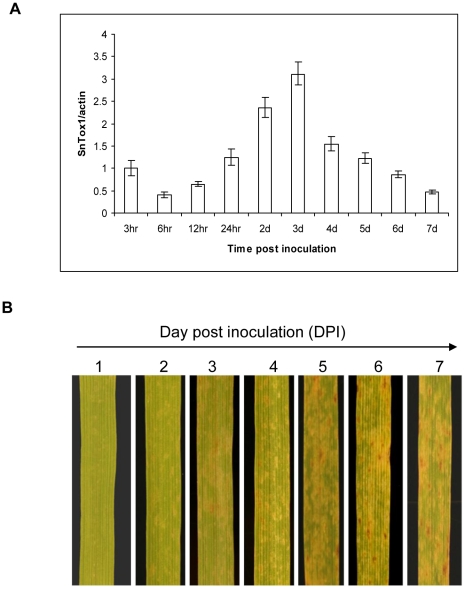
*SnTox1* expression is induced *in planta* during disease development. **A**. Expression pattern of *SnTox1 in planta* during disease development. The x axis shows the time points post-inoculation when leaf samples were taken for qPCR. The y axis represents the relative gene expression levels normalized to *Act1*. Standard error bars from three replications are shown. **B**. Macroscopic examination of disease development on CS inoculated with Sn79+SnTox1A1. The samples of leaves of CS inoculated with Sn79+SnTox1A1were collected and photographed at 24 h intervals post-inoculation.

*SnTox1* had a very similar expression pattern as *SnToxA* and *SnTox3* during infection in a microarray analysis that examined the expression of all fungal genes at 3, 5, 7, and 10 days post inoculation (DPI) in the wheat cultivar ‘Amery’ inoculated with SN15 (Ip-Cho and Oliver unpublished data). The analysis showed that the expression of all three genes was highest at 3 DPI (Figure S5). In this work, *SnTox1* expression was examined after inoculation of CS with *Sn79+SnTox1A1*, in which no other toxin-sensitivity gene interactions were involved. In the current study, relative expression of *SnTox1* to the fungal actin gene was examined at 10 time points ranging from 1 h to 7 d post inoculation using relative-quantitative PCR. Our analysis confirmed that *SnTox1* expression was maximized at 3 DPI (Figure 7A). The expression of *SnTox1* showed a slow increase between 6 and 12 HPI, increasing to about the same level as the actin gene at 24 HPI and increasing dramatically to 2.5 times higher than the actin gene expression at 48 HPI (Figure 7A). Once gene expression peaked at 3DPI, the *SnTox1* transcription levels started to drop significantly from 3 to 4 DPI and returned to similar levels as the actin gene between 5 and 6 DPI. The accelerated increase of *SnTox1* expression from 24 HPI to 3 DPI indicates that *SnTox1* plays an important role in the early stage of infection.

The symptom development was examined macroscopically on CS inoculated with *Sn79+SnTox1A1* (Figure 7B). Disease symptoms were first visible on leaves at 2 DPI as white flecks and progressed into larger necrotic and chlorotic lesions. Interestingly, tan necrotic lesions start to develop at 3 DPI within the chlorotic areas, which correlates with the maximum expression of *SnTox1* (Figure 7A). By 5 DPI, necrotic lesions became evident and the chlorotic areas enlarged (Figure 7B). The overall phenotype of the lesions changed very little from 5 to 7 DPI with only a slight change in size of individual lesions (Figure 7B).

### The SnTox1 protein contains 16 cysteine residues that likely form multiple disulfide bonds and are important for SnTox1 stability

**Figure 8:**
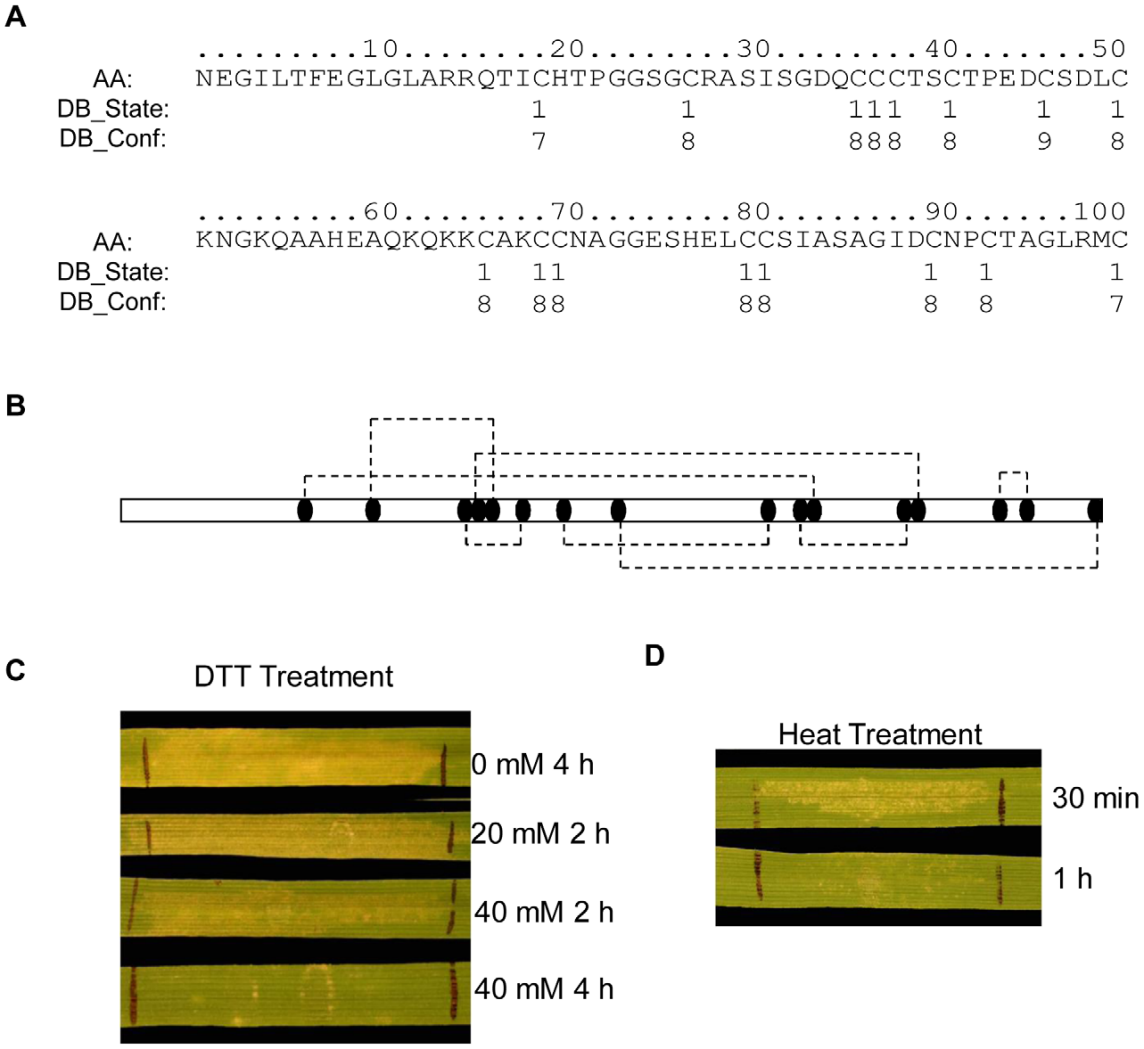
SnTox1 protein is cysteine-rich and highly stable. **A**. Disulfide bond prediction. The prediction was conducted using the web-based program *DISULFIND* (http://disulfind.dsi.unifi.it/). AA: amino acid sequence, DB_state: predicted disulfide bonding state (1 = disulfide bonded, 0 = not disulfide bonded); DB_conf: confidence of disulfide bonding state prediction (0 = low to 9 = high). **B**. The best predicted connectivity of disulfide bonding. The best connectivity of eight disulfide bonds was determined using the web-based program *DiANNA 1.1* (http://clavius.bc.edu/~clotelab/DiANNA/). **C**. Dithiothreitol (DTT) treatment of SnTox1. Reaction of CS to the SnTox1 yeast culture filtrates that were treated with DTT at the indicated concentration for 2 h or 4 h at room temperature. **D**. Heat treatment of SnTox1. Reaction of CS to the SnTox1 yeast culture filtrates that were heated to boiling on a hot plate for 30 min or 1 h.

The SnTox1 protein contains 16 cysteine residues all of which are predicted to be involved in the formation of disulfide bonds with confidence levels greater than 7 (0 to 9 scale, [46]) (Figure 8A). The prediction software DiANNA [47] was used to identify the most likely connectivity of the cysteine residues as following: 1–11, 2–5, 3–6, 4–13, 7–9, 8–16, 10–12, and 14–15 (Figure 8B). The stability of SnTox1 was tested by incubation of an SnTox1-containing yeast culture filtrate with different concentrations of dithiothreitol (DTT) and different incubation periods. The complete elimination of SnTox1 activity required 4 h in 40 mM DTT (Figure 8C). Additionally, the stability of SnTox1 was tested by directly heating the SnTox1 yeast culture filtrates on a hot plate. Strikingly, the culture filtrates maintained necrotic activity even after boiling for 30 min and did not completely lose activity until after 1 h (Figure 8D). Together, these results show that SnTox1 is a highly stable protein with the ability to resist physical and chemical degradation.

### SnTox1 triggers an oxidative burst

**Figure 9:**
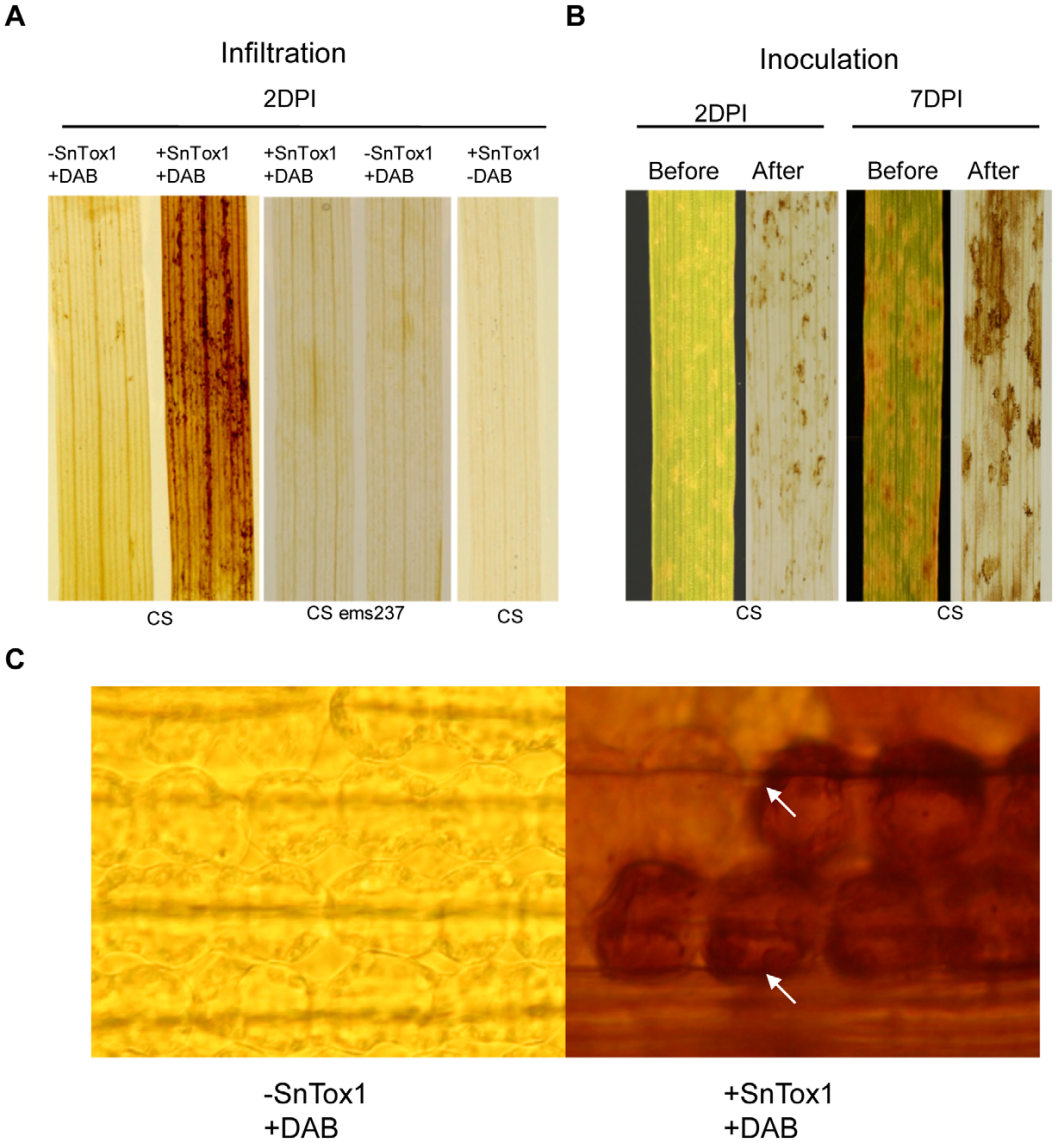
SnTox1 triggers H_2_O_2_ production. **A**. H_2_O_2_ production in infiltrated leaves. CS (*Snn1*) or CS ems237(*snn1*) leaves were infiltrated with SnTox1 (+SnTox1) or control culture filtrates (-SnTox1) and stained with 1 mg/ml DAB (+DAB) followed by clearing of chlorophyll. Leaves infiltrated with SnTox1 were cleared without staining as a control (-DAB) to show that the toxin reaction itself does not cause the brown color. **B**. H_2_O_2_ production in the inoculated leaves. CS leaves were inoculated with *Sn79+SnTox1A1* and collected at each day post inoculation for DAB staining followed by clearing of chlorophyll. Photograph was taken before and after DAB staining and clearing. 2 DPI and 7 DPI are shown. **C**. Location of H_2_O_2_ production in plant cells. The DAB-stained CS leaves were examined under the light microscope for the cellular location of DAB staining. The strong DAB staining was localized to the chloroplasts (white arrows) only in CS infiltrated with SnTox1 (400x magnification).

The oxidative burst is one of the best-known biochemical responses of plant cells during a resistance response. The oxidative burst can be visualized by 3′–3′ diaminobenzidine (DAB) staining for H_2_O_2_ production [48]. Chinese Spring (CS, *Snn1*) wheat leaves were infiltrated with SnTox1 yeast culture filtrate or a control yeast culture filtrate and collected at 48 h post-infiltration. The CS ems237 line (*snn1*) was included for infiltration and DAB staining as a comparison. Leaves were stained with 1 mg/ml DAB solution followed by clearing of chlorophyll. Dense brown DAB staining was observed on the leaves of CS (*Snn1*) infiltrated with SnTox1, but DAB staining did not appear on leaves of CS infiltrated with the control culture filtrates deficient in SnTox1, nor did DAB staining appear when SnTox1 was infiltrated into leaves of CS ems237 lines (*snn1*) (Figure 9A), clearly showing that the production of H_2_O_2_ is induced only during the SnTox1-*Snn1* interaction. A control without DAB staining was also conducted on CS leaves infiltrated with SnTox1 yeast culture. After clearing the leaf, no browning was observed indicating that, in the absence of DAB, the SnTox1 reaction itself was not able to cause brown staining on the leaf (Figure 9A).

The production of H_2_O_2_ was also detected during the fungal infection. The CS leaves inoculated with *Sn79+SnTox1A1* were collected daily from 1 to 7 days post inoculation and stained with DAB followed by the same procedure for leaf clearing. The accumulation of brown staining on the leaf was readily visible from 2 DPI (Figure 9B). The generation of reactive oxygen species (ROS) associated with a hypersensitive response *in planta* often occurs in the chloroplast [49]. Using DAB stained CS leaves from the SnTox1 infiltration, we observed that chloroplasts had the highest intensity of brown color (Figure 9C).

### SnTox1 triggers stronger up-regulation of PR-genes

**Figure 10:**
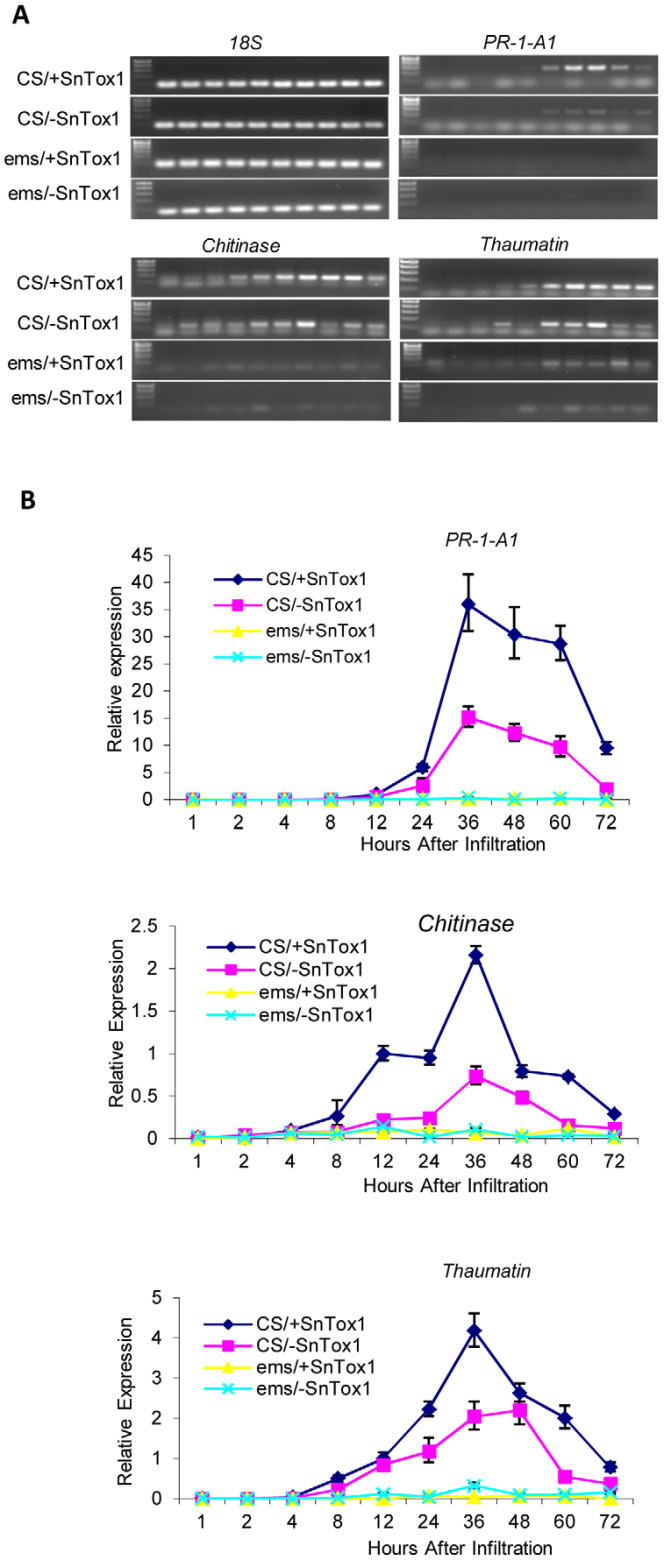
SnTox1-*Snn1* interaction induces increased defense gene expression. **A**. Expression of three pathogenesis-related (PR) genes using RT-PCR. CS (*Snn1*) and CS ems237-1 (*snn1*) leaf samples were collected at 1, 2, 4, 8, 12, 24, 36, 48, 60, and 72 h after infiltration with SnTox1 or control yeast culture filtrates. RT-PCR was conducted to compare the expression of three PR-genes (*PR-1-A1*, *chitinase*, and *thaumatin*) among four different interactions including CS infiltrated with SnTox1 culture filtrates (CS/+SnTox1), CS infiltrated with control culture filtrates (CS/-SnTox1), CS ems237 infiltrated with SnTox1 (ems/+SnTox1), and CS ems237 infiltrated with control culture filtrates (ems/-SnTox1). The wheat 18S gene was used as an RNA quantity control. **B**. Expression of three PR genes using qPCR. Comparisons were made among the four different interactions described above. The same RNA samples from RT-PCR were used in qPCR analysis. The relative expression level for each time point was normalized to the wheat 18S gene.

Up-regulation of plant defense or signaling pathway genes including pathogenesis-related (PR) genes are hallmarks of a resistance response. Using RT-PCR, we examined the transcription level of 28 wheat genes (Table S3) in CS (*Snn1*) and CS ems237 (*snn1*) leaves that were collected at different time points from 1 h to 72 h after being infiltrated with SnTox1 culture filtrates as well as control culture filtrates. Three genes including PR-1-A1, a thaumatin-like protein gene, and a chitinase were found to be significantly up-regulated in CS leaves infiltrated with SnTox1 compared to the control leaf samples infiltrated with culture filtrates deficient in SnTox1 (Figure 10A). In the CS ems237 line which has a mutated *snn1* gene, a transcript was undetectable for the *PR-1-A1* gene and was at a significantly lower level for the *thaumatin* and *chitinase* genes as detected by RT-PCR (Figure 10A). Quantitative PCR (qPCR) analysis confirmed the higher expression of the three genes in SnTox1 infiltrated CS leaves compared to the control infiltrated CS leaves. Not only did all three genes show maximum expression at 36 HPI, but each had at least two-fold higher expression in SnTox1-infiltrated samples than the control (Figure 10B). qPCR also showed much lower expression of the three genes in the CS ems237 line infiltrated with either SnTox1 or the control yeast culture filtrates in comparison to CS infiltrated with control culture filtrates (Figure 10B). The reason for this is not clear, but it could be explained by the idea that *Snn1* may play a role in sensing other environmental stimuli that can trigger PR gene expression.

### The SnTox1-*Snn1* interaction triggers DNA laddering

**Figure 11:**
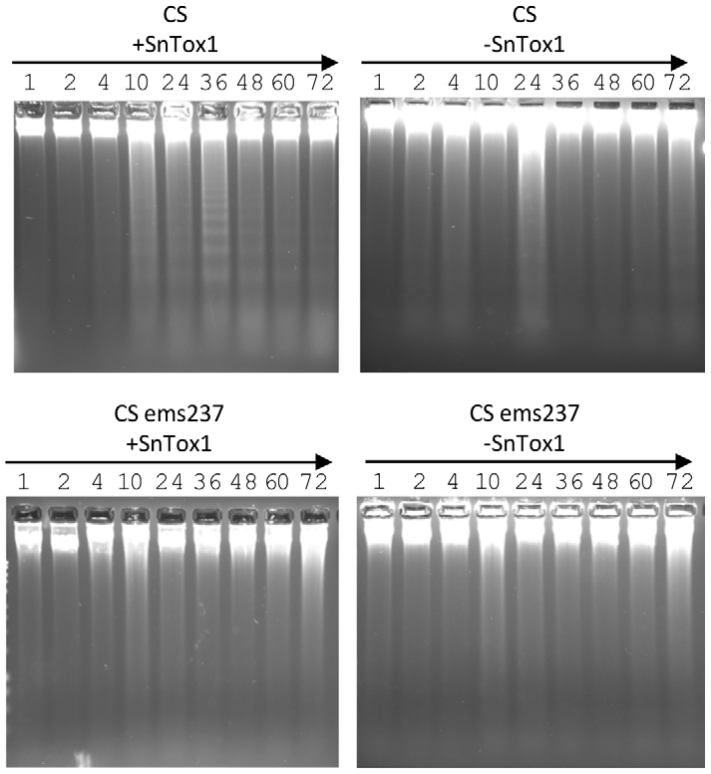
The SnTox1-*Snn1* interaction induces programed cell death. Leaf samples of Chinese Spring (CS) and CS ems237 were collected at 1, 2, 4, 10, 24, 36, 48, 60 and 72 h post infiltration with SnTox1 yeast culture filtrates (+SnTox1) and control culture filtrates (-SnTox1). Genomic DNA was extracted from the collected leaf samples, run on a 2% agarose gel and stained with ethidium bromide. DNA fragmentation was detected only in leaves of CS infiltrated with SnTox1 culture filtrates, but not in leaves of CS infiltrated with control culture filtrates, CS ems237 infiltrated with SnTox1, or control culture filtrates.

Programmed cell death (PCD) triggered by biotrophic effectors is often evidenced by DNA laddering in plants [16], [50]. To determine if the necrosis induced by SnTox1 on *Snn1* lines was a result of PCD, we isolated DNA from infiltrated CS leaf samples and checked for evidence of DNA laddering. For negative comparisons where no necrosis developed, DNA fragmentation was also examined in CS leaves infiltrated with control culture filtrates (no SnTox1) and CS ems237 (mutated *snn1*) infiltrated with SnTox1 or control culture filtrates. In the CS leaf samples infiltrated with SnTox1, DNA laddering was detected as early as 10 h after infiltration and was most evident at 36 h after infiltration (Figure 11); however, in the leaf samples from the other three treatments, no DNA laddering was observed at any time point (Figure 11), indicating that SnTox1-induced necrosis on lines harboring *Snn1* is a result of host-controlled PCD.

### Light is required for the development of necrosis and disease induced by SnTox1

**Figure 12:**
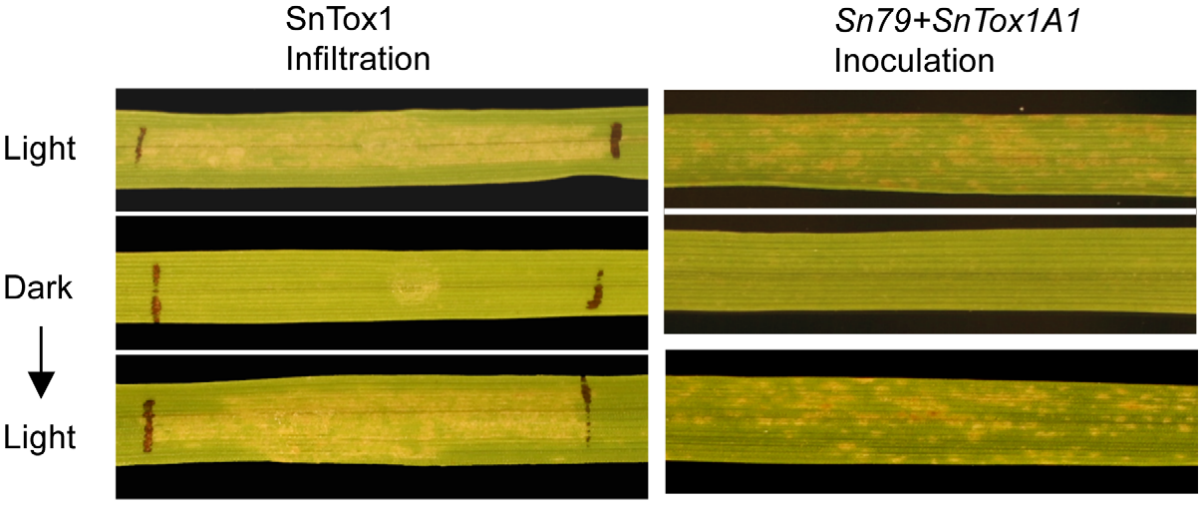
The SnTox1-*Snn1* interaction is light dependent. The light dependency of the SnTox1-*Snn1* interaction was tested in both infiltration (left panel) and inoculation (right panel) experiments. Chinese Spring plants (*Snn1*) were grown under a normal light-dark cycle (12 h photoperiod) or under complete dark conditions for 48 h after being infiltrated with SnTox1 culture filtrates or inoculated with conidia from Sn79+SnTox1A1. No necrosis or lesions developed in the plants under dark treatment in either experiment, however, once the dark treated plants were moved back to a normal light cycle, necrosis and lesions developed subsequently on the leaves after an additional 48 h.

Light has been found to be important in the development of necrosis induced by necrotrophic effectors from *P. tritici-repentis* and *S*. *nodorum* [12], [37]. Therefore, we investigated whether the development of necrosis induced by SnTox1 as well as the disease development caused by the SnTox1-*Snn1* interaction was light dependent. After infiltration with SnTox1 yeast culture filtrates, CS plants were incubated in a growth chamber but covered for 2 days. The plants in the dark did not exhibit a necrotic reaction in the infiltrated area on the leaves, while the plants grown in the same growth chamber without covering showed necrosis (Figure 12) indicating the development of necrosis induced by SnTox1 is light dependent. Interestingly, necrosis did develop on the dark treated plants once they were treated with a 12 h light-dark cycle for 2 additional days.

A very similar situation was observed in the inoculation experiment. CS leaves showed no disease symptoms at 3 days post inoculation when plants were kept in the dark and similar to the infiltration experiment, the lesions developed once the dark-treated plants were moved to the light again (Figure 12).

### SnTox1 has an important function during the penetration period

**Figure 13:**
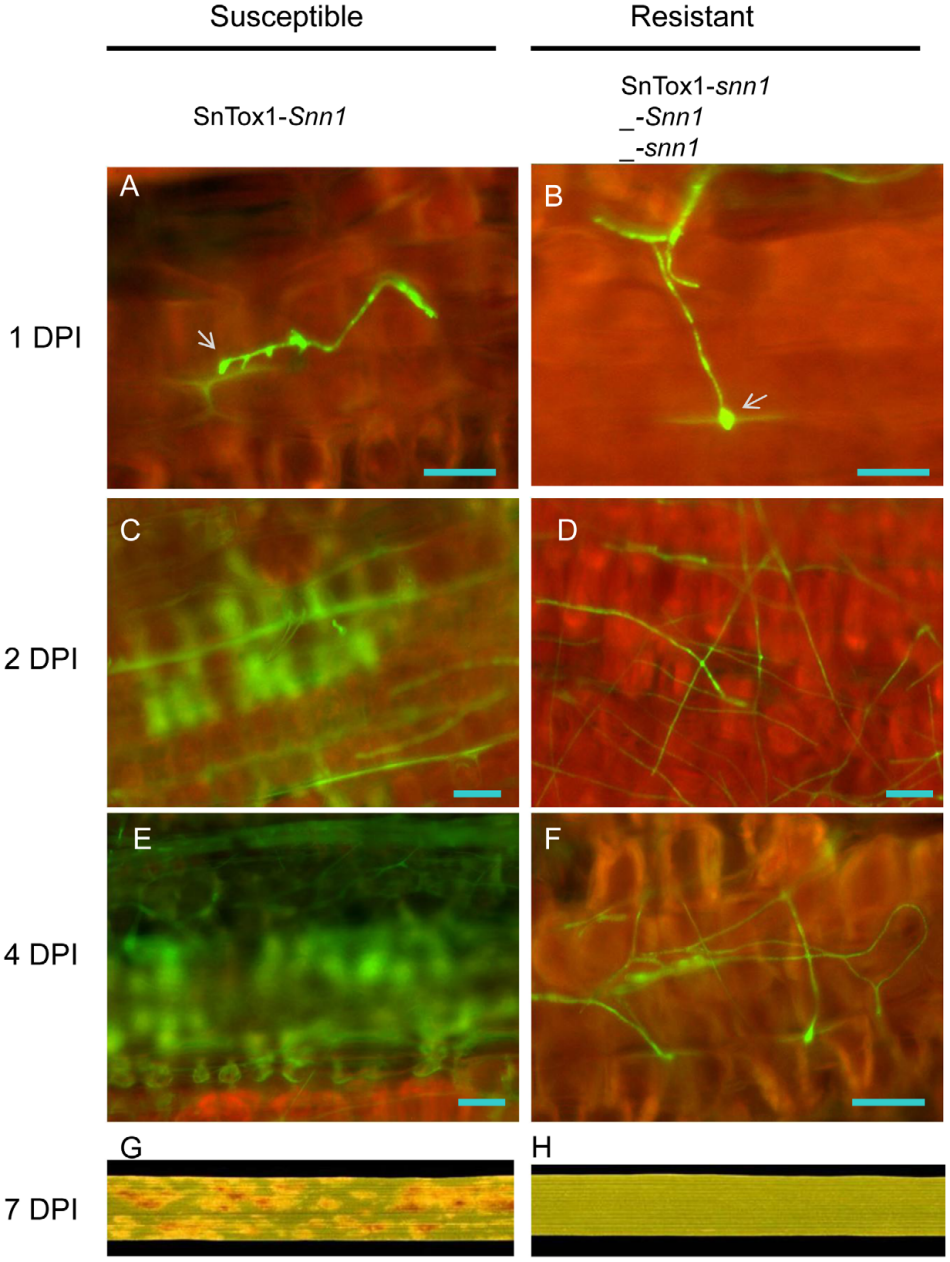
Comparison of the fungal infection process with or without an SnTox1-*Snn1* interaction. The fungal infection process was examined and compared at 1, 2, 4 and 7 days post inoculation (DPI) between the susceptible interaction (**A, C, E, G**) and resistant interaction (**B, D, F and H**) using GFP-tagged fungal strains and confocal fluorescent microscopy. **A, B.** Infection at 1 DPI. The fungus was able to form penetration structures (hyphopodium, white arrow) in both interactions. **C, D,** Infection at 2 DPI. The fungus breached the epidermal layer and started infecting the mesophyll layer in the susceptible interaction shown by the autofluorescence (C). No autofluorescence was observed in the resistant interaction (D). **E, F,** Infection at 4 DPI. The SnTox1+ strain continued infecting mesophyll cells shown by the larger area having autofluorescence in the susceptible interaction (E), but the SnTox1- strain remained on the leaf surface and continued attempting to penetrate in the resistant interaction (F). **G, H,** infection at 7DPI. The disease lesions have fully developed in the susceptible interaction (G), but no disease symptom was observed in the resistant interaction (H). Scale bar  = 20 µm.

To investigate the role of SnTox1 in disease development, we tagged both the avirulent isolate Sn79-1087 and the pathogenic strain *Sn79+SnTox1A1* with GFP and compared their infection processes by fluorescence microscopy in wheat lines CS (*Snn1*) and the *Snn1* mutant, CS ems237 (*snn1*). The inoculation of CS with the SnTox1-producing strain Sn79+SnTox1A1 resulted in an infection (susceptible interaction); however, the other three combinations (CS inoculated with Sn79-1087, CS ems237 inoculated with Sn79-1087, and CS ems237 inoculated with *Sn79+SnTox1A1*) gave no disease (resistant interaction) (Figure 13). Within 24 HPI, there was little difference observed between resistant and susceptible interactions. During this period, conidia germinated, grew short hyphae and began the penetration process. The pathogen was able to initiate penetration in both types of reactions visualized by the formation of the indistinct penetration structure called a hyphopodia [26]; Figure 13 A, B) and by autofluorescence of the damaged epidermal cell walls (Figure 13 A, B). We observed mainly direct penetration of the leaf surface over both periclinal and anticlinal epidermal cell walls.

A strong green autofluorescence was observed beneath the epidermis by 2 DPI in the susceptible interaction, suggesting that the pathogen had successfully penetrated through the epidermal cell layer and started the infection of mesophyll cells (Figure 13C). However, in the resistant interaction, the pathogen grew extensively on the leaf surface and no green autofluorescence was visible (Figure 13D). At 4 DPI, the infection area had enlarged in the susceptible interaction as shown by more mesophyll cells producing a fluorescent signal (Figure 13E). On the leaves of the resistant interactions, most of the fungal mycelium was dead, likely due to scarcity of nutrients, and only a few hyphae continued to grow over the leaf surface with repeated unsuccessful attempts to penetrate (Figure 13 F). The susceptible interaction had induced widespread lesion formation on the leaves by 7 DPI, however, no symptoms were found on the leaves of the resistant interaction (Figure 13 G, H). Examination under the fluorescent microscope of the necrotic lesion formed from the susceptible reaction revealed the extensive growth of fungal mycelium within the lesion (Figure S6).

### Light is required for penetration during infection

**Figure 14:**
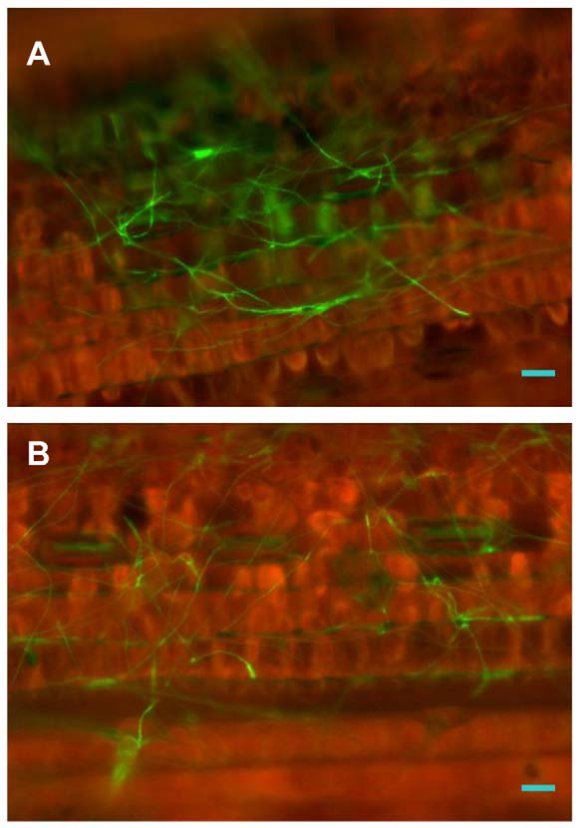
Light is required for penetration of the epidermis. The fungal infection process was examined and compared between plants grown under a normal light cycle and those under complete darkness post inoculation. At 48 HPI, the pathogen successfully penetrated through the epidermal cell layer and started infection of mesophyll cells in the CS leaves that were grown under a normal light cycle (A), but not in the leaves that were grown in the dark (B). Scale bar = 20 µm.

The fungal infection process was also compared microscopically on *Snn1*-containing plants that were either grown under a normal light/dark cycle or in complete darkness after inoculation. The pathogen was able to germinate and generate hyphopodia within 24 HPI in both conditions (data not shown). However at 48 HPI, only the plants grown in a normal light/dark cycle showed successful penetration through the epidermal cell layer and the initiation of the infection of mesophyll cells, evidenced by the autofluorescence of the mesophyll cells (Figure 14A). In the plants that were kept in the dark, no autofluorescence was observed in the mesophyll cells and the pathogen still remained on the leaf surface without having successfully penetrated the epidermis (Figure 14B).

## Discussion

### Molecular cloning of *SnTox1*

The necrotrophic fungal pathogen *S. nodorum* produces multiple necrotrophic effectors (host-selective toxins) that function as virulence factors during the infection process. The cloning of these necrotrophic effector genes is an essential step in the characterization and elucidation of the molecular and biochemical mechanism of fungal pathogenesis in the wheat-*S*. *nodorum* pathosystem. Besides the traditional biochemical and genetic tools, new genomic strategies have been recently applied for the identification and cloning of effector genes in a number of fungi and oomycetes as more genome and other sequence data becomes available. A typical procedure would include a process of data mining to identify candidate genes that meet a set of specific criteria followed by gene validation through functional analysis. High throughput functional genomics [1] as well as comparative genomics and association genetics [41] have been successfully used for the identification of pathogen effector genes in fungi and oomycetes. In the current study, we used a set of criteria to mine the *S*. *nodorum* genome sequence dataset for the identification of necrotrophic effector genes. This strategy led to the successful identification of *SnTox1* from *S*. *nodorum*. Through heterologous expression, gene transformation, and gene disruption, we have provided convincing evidence that the candidate gene *SNOG20078* (Gene ID: 5974395) is the SnTox1-encoding gene. This research further highlights the value of genome sequence data along with efficient bioinformatics tools in identifying effector genes. We are continuing to use this strategy to identify additional *S. nodorum* necrotrophic effector genes.

### The genomic location of *SnTox1*

*SnTox1* was identified using a set of criteria based on the cloned *S*. *nodorum* effector genes *SnToxA* and *SnTox3*; however, the *SnTox1* gene does have some unique features. Unlike many previously identified effector genes including those from *Leptosphaeria maculans* [51]–[53], *Magnaporthe grisea* [41], [54], *Fusarium oxysporum* f. sp. *lycopersici* [55], [56], *Blumeria graminis* f. sp. *hordei* [57], and those from several *Phytophthora* species [58], *SnTox1* lies in a gene-rich region and was flanked closely by other genes. Except for a short (≈300 bp) sequence predicted to be a truncated molly-type RE, no other obvious RE or AT-rich sequences were identified within the 300 kb genome region surrounding *SnTox1* (http://genome.jgi-psf.org/Stano1/Stano1.info.html) showing that not all effector genes are associated with an abundance of repetitive or transposable elements.

The occurrence of effector genes in close proximity to one another has also been reported for several fungal and oomycete pathogens [53], [59]–[62]. This does not appear to be the case for *S. nodorum*. The three *S*. *nodorum* effector genes (*SnToxA*, *SnTox1*, and *SnTox3*) were located on different supercontigs and have been shown by pulse field gel electrophoresis and Southern analysis to reside on 2.35, 1.88 and 1.66 Mb chromosomes, respectively, in SN15 (data not shown) indicating that these genes are not clustered.

### The population genetics of *SnTox1*

Using a worldwide collection of 777 *S. nodorum* isolates, *SnTox1* was found to be present in ∼85% of isolates, a markedly higher frequency than found for SnToxA (∼36%) and SnTox3 (∼60%) [14], [63]. Like the other NEs, *SnTox1* was shown to have a presence/absence polymorphism within individual wheat fields. This type of polymorphism has been reported in other fungal pathosystems, as reviewed in Stergiopoulos and de Wit [64]. The frequency of *SnTox1* varied significantly across regional populations. We hypothesize that regional differences in the frequency of *SnTox1* reflect regional differences in the frequency of *Snn1*. However this correlation was not apparent when tested on a small worldwide collection of wheat. We found that *Snn1* is most prevalent in durum wheat lines and much more rare among hexaploid bread wheat lines throughout the world (data not shown). This could indicate that the maintenance of *Snn1* in durum wheat is associated with another important trait. Widespread deployment of wheat cultivars lacking *Snn1* could cause the frequency of *SnTox1* to decrease if there is a fitness cost associated with producing the effector. But the large effective population sizes of *S. nodorum* [65] make the complete loss of the effector through genetic drift unlikely.

Observed diversity at the *SnTox1* locus was found to fit a model of diversifying selection significantly better than a neutral model. Positive selection was found for 4 of the *SnTox1* codons, consistent with the growing list of prokaryotic and eukaryotic effector candidates that exhibit positive selection [66]. None of the non-synonymous substitutions were found in the signal peptide, the putative chitin-binding domain, the putative Avr4-like domain or any of the cysteine codons. This suggests that the effector's functional domains were preserved, while more flexible amino acid sites were subject to diversifying selection. Possible differences in activity between different protein variants of SnTox1 are currently being tested.

### SnTox1 is a small secreted and cysteine rich necrotrophic effector

Similar to *SnToxA* and *SnTox3*, *SnTox1* was shown to play a significant role in disease development. Results presented here on the SnTox1-Snn1 interaction provide further evidence that the necrotrophic wheat-*S*. *nodorum* system is largely based on specific host-effector interactions that act in ETS [14], [15], which essentially has the opposite outcome of ETI that operates in many biotrophic systems [3], [4].

One of the most striking features of the SnTox1 protein as a necrotrophic effector is the high cysteine residue content. This feature is often associated with fungal avirulence gene products such as the Avr and ECP effectors from *Cladosporium fulvum* [64], SIX (secreted in xylem) effectors from *Fusarium oxysporum* f.sp. *lycopersici* [55], and Nip1 from *Rhynchosporium secalis* [67]. The predicted mature SnTox1 protein has 100 amino acids, 16 of which are cysteine residues, the richest of all effectors that have been identified. The high content of cysteine residues and high stability suggest that SnTox1 may function in the plant apoplastic space which is abundant in plant defense components. We are currently investigating the subcellular location of SnTox1. Most small cysteine-rich secreted effectors from the tomato fungal pathogen *C*. *fulvum* such as Avr2, Avr4, Avr9, and ECP2 are thought to function exclusively in the apoplast to inhibit and protect against plant hydrolytic enzymes [64]. ECP6, another *C*. *fulvum* effector containing LysM chitin binding domains was recently found functioning apoplastically as a scavenger of fungal chitin to prevent it from eliciting PAMP-triggered immunity *in planta* [68]. Interestingly, we observed that SnTox1 has some similarity to *C. fulvum* Avr4 within the chitin-binding domain and in the positions of six of the cysteine residues at the C-terminus. However, further tests are needed to determine the binding activity and functional roles of the putative CB domain in SnTox1. The presence of a potential chitin binding domain provides a point of investigation for an added function for SnTox1 in addition to its interaction with *Snn1*.

### The SnTox1-*Snn1* interaction enables direct penetration

Successful penetration is a prerequisite for a pathogen to establish itself and fulfill its colonization *in planta*. For *S*. *nodorum*, previous studies have observed direct penetration through the junction of epidermal cells [69] or penetration through stomata [70] or both [26]. Based on our observation using GFP-tagging and confocal fluorescent microscopy, the fungus predominantly used direct penetration through the junction of epidermal cells, and stomatal entry was not evident. We have observed that the fungal mycelium grew over guard cells and anchored the penetration point between the junction of the guard cell and the adjacent epidermal cell instead of going through the stomata (data not shown). Although the avirulent isolate belongs to *S. nodorum*, the preference for direct penetration, which is different from that reported by Solomon et al. [26], may be due to its adaptation to wild grasses from which it was originally isolated.

It was our observation that the fungus could initiate direct penetration by producing a hyphopodia in both the resistant and the susceptible interaction with little difference, which agrees with previous reports [69] indicating that SnTox1 is not required for hyphopodia formation and the initial degradation of the cuticle layer and the cell wall between the junctions of the epidermal cells. Hydrolytic enzymes or other mechanisms may be employed by the fungus to breach the initial physical barrier. Several cell wall-degrading enzymes such as amylase, pectin methyl esterase, polygalacturonases, xylanases, and cellulase have been found to be produced *in vitro* and during the infection of wheat leaves by *S*. *nodorum* [71]. As infection progressed, the pathogen was unable to penetrate through the epidermal cell layer and therefore could not reach the mesophyll cells to establish a successful infection without the SnTox1-*Snn1* interaction. This suggests that SnTox1 is significant in the initial penetration process across the epidermal cell layer. Our hypothesis is that SnTox1 interacts with *Snn1* to induce cell death in epidermal cells, providing the fungus with nutrients for further invasive growth. In *Cochliobolus victoria* on oat and *Arabidopsis* systems, it was also observed that fungal penetration ceases following appressorium development and hyphae remain on the leaf surface in the absence of a compatible interaction, which requires both victorin and its corresponding sensitivity gene [22]. Our speculation was further supported by the fact that the inoculation of an *Snn1* line (CS) with *SnTox1* transformed avirulent isolates induced widespread necrosis - presumably programmed cell death - on leaves. Furthermore, inoculation with the *SnTox1*-knock out virulent strain lost the ability to cause this necrotic reaction. Additionally, qPCR revealed that *SnTox1* expression was induced *in planta* starting as early as 12 HPI and increased at an accelerated rate from 12 to 24 HPI when the fungus was observed to penetrate. Collectively, this suggests that *S. nodorum* may use SnTox1 to induce cell death in the epidermal cells, providing a portal to enter the plant and subsequently feeding from dead cells to gain nutrients for further invasive growth.

### SnTox1 induces a light dependent reaction

It is well known that plant defenses against pests and pathogens are commonly influenced by environmental conditions, including light. Many studies have demonstrated the requirement of illumination for the interaction of plants with a diversity of bacterial and fungal pathogens as well as the isolated pathogenic elicitors [72], [73]. The effect of light on the disease development of SNB was first noticed by Baker and Smith [69] who observed that the necrotic reaction and lesion coalescence tended to be suppressed in the absence of light. The necrotrophic effector ToxA, was also shown to induce light-dependent necrosis on *Tsn1* lines [37]. Among the *S*. *nodorum* necrotrophic effectors published to date, all effectors except SnTox3 have been shown to be light dependent [13], [32]. Using heterologously expressed SnTox1 and the avirulent isolate carrying the *SnTox1* gene, we showed clearly that the necrosis and disease susceptibility induced by SnTox1 on *Snn1* lines were completely dependent on light. The requirement of light for resistance to biotrophic disease as well as susceptibility to necrotrophic disease suggests a common host mechanism shared by reactions to the two classes of disease interactions.

The molecular mechanism underlying the light dependency of plant pathogen interactions is still poorly understood; however, research on the ToxA-*Tsn1* interaction has shown that ToxA is internalized in the plant cell followed by localization to the chloroplast and induction of photosystem alterations (reviewed in [40]), providing a hint for the influence of light on this interaction. Recently, it was demonstrated that *Tsn1* is regulated by light and its expression is significantly suppressed in the dark [15], providing a possible explanation for the light dependency of the ToxA-*Tsn1* interaction. SnTox1 is cysteine rich and therefore possibly acts in the apoplastic space. If SnTox1 remains in the apoplastic space, different mechanisms would likely be involved even though both are dependent on light. In Arabidopsis, plants kept in the dark do not accumulate H_2_O_2_ in the chloroplasts and show significantly delayed HR cell death after a resistance signaling pathway is activated [49]. This indicates that light is required for H_2_O_2_ production in chloroplasts and that this H_2_O_2_ production is critical to programmed cell death. The DAB staining in CS (*Snn1*) leaves infiltrated with SnTox1 was found to be associated with the chloroplast and the CS plant infiltrated with SnTox1 showed no DAB staining if kept in the dark (data not shown), suggesting a similar mechanism underlying SnTox1-induced cell death. Very interestingly, we found that plants kept in the dark developed necrosis and disease symptoms once transitioned to a normal photoperiod. Therefore signal transduction appears to pause rather than stop in the absence of light. This may indicate that the SnTox1 signal is progressing to the chloroplast but this process is interrupted in the absence of light.

### SnTox1 functions like a biotrophic effector to induce PCD in *Snn1* host lines

Biotrophic effectors often function as elicitors of programmed cell death (PCD) thereby activating the resistance response in host plants containing the corresponding resistance genes. The host resistance reaction begins with the direct or indirect recognition of the pathogen-produced effector by the resistance gene product, followed by a complicated signaling pathway and a series of biochemical and physiological responses in host plant cells [74]. The host response often includes an oxidative burst, cell wall restructuring, PR-gene expression and antimicrobial compound production culminating in a localized cell death at the infection site. This PCD is known as a hypersensitive response and is typically aimed at halting further colonization by the pathogen [5]. A set of biochemical tests has shown that SnTox1 is able to induce resistance-like host responses and PCD evidenced by the H_2_O_2_ production, stronger expression of PR-genes, and DNA laddering in lines carrying *Snn1*. It is important to note that SnTox1 physiologically evoked a widespread necrotic flecking on the *Snn1* line, which is symptomatically similar to the hypersensitive response in biotrophic disease systems. However, this necrosis spreads into larger lesions resulting in susceptibility (sporulation) rather than resistance (prevention or inhibition of sporulation). Together, this indicates that SnTox1 is likely functioning biochemically and physiologically similar to a biotrophic effector (avirulence factor) in the presence of *Snn1* but with a different end result.

A number of other necrotrophic effectors have also been shown to invoke a host resistance response [9], [17], [40]. It has generally been thought that necrotrophic plant pathogenic fungi possess simplistic infection mechanisms that rely on lytic and degrading enzymes [11]. In contrast, biotrophic fungal pathogen interactions have been considered more sophisticated due to the formation of special penetration and feeding structures, secretion of effectors to overcome plant PAMP triggered immunity and a constantly changing effector complement to avoid recognition by the plant innate immune system. However, three genes conferring sensitivity to necrotrophic effectors as well as susceptibility to the corresponding necrotrophic fungal pathogens have been cloned, and all possess resistance gene-like features [15], [22], [23]. Therefore, it seems that necrotrophic fungal pathogens may subvert plant resistance mechanisms for their own good. Here, we clearly showed that SnTox1 is an important virulence factor for *S. nodorum* in the presence of *Snn1* and that the host response to SnTox1 shows several similarities to a classical resistance response induced by many biotrophic effectors, however, the outcome of the host recognition was susceptibility rather than resistance.

*SnTox1* is the third effector gene that we have cloned and characterized from *S. nodorum*, which further strengthens the hypothesis that the wheat-*S. nodorum* pathosystem is based largely on host-effector interactions. The three effector genes cloned have provided molecular tools to study the mechanisms underlying disease in this system, an emerging model for necrotrophic fungal diseases.

## Materials and Methods

### Bioinformatics for prioritizing candidate genes

A series of experimental and bioinformatic criteria associated with effectors were evaluated to produce a candidate gene ranking of the predicted genes in the *S*. *nodorum* genome. These criteria were based on the known and predicted properties of effectors. Genes matching different criteria were given scores from 1 to 6. The sum of scores for each gene was ranked and the top 100 genes were considered. The criteria used data from mass-spectrometry analyses of culture filtrates, a genome sequence scan of the strains Sn4 and Sn79-1087, an *in planta* microarray experiment and various bioinformatics analyses. The criteria were as follows: predicted to be less than 30 kDa (1 point), cysteine rich (>1 standard deviation more cys residues than expected of a protein of that size) (2 points), detected by MS in culture filtrates (6 points), located within 5 kb of repetitive sequences (2 points), absence of homologues in the NCBI nr database (2 points), presence of RXLR or RGD motifs (2 points), predicted to be secreted (3 points), presence of a modified version of the gene in Sn4 (3 points), absence of the gene in Sn79-1087 (4 points), and a gene expression profile similar to ToxA and Tox3 (3 points).

### Yeast expression of SNOG_20078 for verification that it was *SnTox1*

The total RNA of 7 day old mycelium of SN15 grown in Fries media [27] was prepared using the RNeasy plant mini kit (Qiagen) and treated with RNase-free DNase I (Promega). First-strand cDNA was synthesized from 2 µg of total RNA using TaqMan Reverse Transcription Reagents (Applied Biosystems). The coding region of *SNOG_20078* was amplified from the above cDNA sample using primers 20078CF_EcoRI and 20078CR_ApaI containing the indicated restriction site (Table S4). The cloning of *SNOG_20078* into the corresponding sequencing and expression vectors, yeast transformation, and preparation of culture filtrates from yeast cultures all followed the procedure described by Liu et al. [14]. The pGAPZ A vector containing the *SNOG_20078* gene was linearized with *Avr*II before transformation. Culture filtrates of the yeast culture transformed with the *SNOG_20078* coding region were infiltrated into wheat lines including BR34 (*snn1*), Grandin (*snn1*), BG220 (*snn1*), BG223 (*snn1*), BG261 (*snn1*), W-7984 (*Snn1*), Chinese Spring (*Snn1*), Opata85 (*snn1*), and ND495 (*snn1*). Because the culture filtrates caused necrosis on W-7984 and CS, which both possess *Snn1* [27], it was infiltrated onto CS 1BS-18, CS ems237, and the ITMI population [27] for verification of SnTox1 based on its interaction with *Snn1*. CS 1BS-18 carries a deletion in the distal end of chromosome 1B that harbors the *Snn1* locus [27]. CS ems237 is an SnTox1 insensitive mutant derived from CS by EMS (ethane methyl sulfonate) mutagenesis (Faris et al., unpublished data).

### Generation of an SnTox1 antibody and western blot analysis of SnTox1

A 14 amino acid long peptide (sequence: CKNGKQAAHEAQKQ), designated SnTox1:50–63, was synthesized by GenScript (Piscataway, NJ). The peptide SnTox1:50–63 (4.7 mg, 0.003 mmole) was first conjugated to bovine serum albumin (BSA, 20 mg, 0.0003 mmole, Sigma-Aldrich, St Louis, MO) in the presence of 1-ethyl-3-(3-dimethylaminopropyl)-carbodiimide hydrochloride (EDC, 20 mg, Pierce Biotechnology, Rockford, IL) in 2 mL of 100 mM 2-(N-morpholino) ethanesulfonic acid buffer, pH 6 overnight at 4°C. The protein was separated from EDC through a size-based column (D-Salt Excellulose, Thermo Scientific, Rockford, IL) and concentrations were determined by the method of Bradford (Bio-Rad Laboratories, Inc. Hercules, CA) using BSA as the calibration standard. Success of the conjugation reaction was assessed on a 13% SDS-PAGE gel. One hundred milligrams of the immunogen were immunized into New Zealand White Rabbits at 3 week intervals for a total of six immunization cycles. The final sera were collected eight days after immunization and were used for western blot analysis.

To prepare the SnTox1 protein sample for western blot analysis, 5 mL of culture filtrate from an SnTox1 yeast culture and control yeast culture (yeast strain transformed with an empty vector) was precipitated by adding 20 mL of methanol and incubating in a -20 freezer overnight. After centrifuging for 10 min at 13,000 rpm on a HERMLE Z 323K centrifuge with a 220.80 V02 rotor (Labnet), the pellet was retained, air dried and re-suspended in 500 µL of a 1× sample loading buffer. Protein gels were loaded with 50 µL of the resulting sample solution. SDS-PAGE, protein transferring, and color development followed a routine protocol described in Meinhardt et al. [36]. To ensure the quality of protein sample preps, the same amount of sample solution was also run on a gel and visualized by coomassie blue staining.

### Amplification of full-length transcript of *SnTox1*

The same RNA extracted from SN15 was used to amplify the 5′ and 3′ ends of the cDNA of *SnTox1*. The 5′ and 3′ RACE were performed using the Smart RACE cDNA amplification kit (Stratagene, LaJolla, CA) according to the instructions in the user manual with gene-specific primers 20078CF and 20078CR (Table S4). The procedure described by Liu et al. [14] was followed for the cloning and sequencing of the amplified 5′ and 3′ RACE fragments. The obtained sequences from 5′ and 3′ RACE fragments were used to assemble the full length cDNA and determine the 5′ and 3′ UTRs based on the SN15 genome sequence.

### Searches for SnTox1 homologs and protein alignments

SnTox1 and Avr4 homologs were identified from the NCBI non-redundant (nr) protein database (http://www.ncbi.nlm.nih.gov/BLAST/) using BLAST searches. The chitin-binding domains of Avr4 and its homologues were identified using Reverse Position-Specific (RPS)-BLAST searches (www.ncbi.nlm.nih.gov/Structure/cdd/wrpsb.cgi). Amino acid alignments were performed using the MegAlign programs from Lasergene 8.1 software (DNASTAR Inc. Madison, WI). Three-dimensional (3D) structure-based sequence alignment of the putative chitin-binding motifs identified in SnTox1 with those of ChtBD1 and ChBD2 proteins were performed following the previously published data on related structures [43], [44], [75].

### Analyses of *SnTox1* diversity

*SnTox1* presence and absence was screened in 777 *S. nodorum* isolates from seven geographical regions: Australia, Central Asia, East Asia, Europe, Middle East, North America, South America and South Africa (Table S1) using PCR with primer pair Tox1F_Coding and Tox1R_Coding (Table S4). A secondary PCR screen using the conserved primer pair Tox1_XF and Tox1_XR (Table S4) was conducted to confirm questionable PCR amplicons. PCR amplification was performed in 20 µl reactions containing 0.05 µM of each primer (supplied by Microsynth), 1X Dream Taq Buffer (Fermentas), 0.4 µM dNTPs (Fermentas) and 0.5 units of Dream Taq^TM^ DNA polymerase (Fermentas). The PCR cycle parameters were: 2 min initial denaturation at 96°C followed by 35 cycles of 96°C for 30 s, 58°C for 45 s and 72°C for 1 min. A final 5 min extension was made at 72°C. To demonstrate the wide distribution of *SnTox1*, a subset of the global collection (79 isolates), along with 10 avirulent isolates and several related fungal species including Pti2 (*P. tritici-repentis*), ND89-19 (*P*. *teres* f. *teres*), Sm15A (*P*. *bromi*) and S. tr 9715 (*M. graminicola*) (Table S2) were evaluated in a dot blot analysis. For dot blot analysis, the DNA of fungal samples was isolated using a BioSprint 15 instrument (QIAGEN) with the corresponding kit (QIAGEN). The DNA samples were blotted onto a nylon membrane using a Bio-Dot microfiltration apparatus (BIO-RAD) following the instructions in the user manual. The entire *SnTox1* coding region was PCR amplified from the genomic DNA of SN15 and used as a probe for Southern blot analysis. Probe preparation, DNA hybridization, membrane washing and image acquisition followed the protocol described by Faris et al. [76]. The membrane was stripped and hybridized to the *S*. *nodorum* actin gene probe to ensure the quality for all the DNA samples.

Sequences for the entire coding region were obtained using the primer pair Tox1UTR_F and Tox1UTR_R and the primer pair Tox1Fout and Tox1Rout (Table S4). In cases of poor amplification primer pair Tox1F_Coding and a new conserved reverse primer, Tox1R_Conserved (Table S4), were used to confirm observed sequence variation. Sequencing reactions were conducted in 10 µl volume using the BigDye Terminator v3.1 Sequencing Standard Kit (Applied Biosystems) with both the forward and the reverse primer. The cycling parameters were 96°C for 2 min followed by 55 or 99 cycles of 96°C for 10 s, 50°C for 5 s and 60°C for 4 min. The products were cleaned with the illustra Sephadex G-50 fine DNA Grade column (GE healthcare) according to the manufacturer's recommendations and then sequenced with a 3730x/Genetic Analyzer (Applied Biosystems). Alignment of forward and reverse sequences for each isolate was performed in SeqScape software V2.5 (Applied Biosystems, Foster City, CA). Translation and identification of protein haplotypes was also performed using this software.

### Tests for positive selection in *SnTox1*

Codeml implemented in the software PAML (http://abacus.gene.ucl.ac.uk/software/paml.html) was used to test for positive diversifying selection [77]. The program uses four different codon substitution models implemented in a maximum-likelihood framework to test which model, neutral or selection, best fits the data. Each model assumes a different range of values for the estimated value ω (the ratio of non-synonymous to synonymous nucleotide substitutions). Under purifying selection, non-synonymous substitutions are expected to be rare, thus ω will remain below 1. If non-synonymous mutations offer a selective advantage, they will be fixed at a higher rate than synonymous mutations and ω will be greater than one. We compared the null model M1a (neutral), which assumes two site classes, purifying (0<ω_0_<1) or neutral (ω_1_ = 1) to the alternative model M2a (selection), which adds another class of diversifying sites (ω_2_>1). We also compared the more complex null model M7 (neutral) that assumes a beta distribution for 0<ω<1, with the alternative model M8 (selection) which also assumes a beta distribution and adds an additional site class with ω_2_>1. A likelihood ratio test was used to compare the likelihood estimate scores. The model simultaneously calculates the posterior probability for each codon that belongs to a particular site class (e.i. ω>1). If the posterior probability for a codon is high and it belongs to the site class with ω>1, positive selection can be inferred for that codon, known as Bayes Empirical Bayes [78].

### Investigation of the genomic region of *SnTox1* in different *S*. *nodorum* isolates

Based on the annotated SN15 genome sequence, four genes *SNOG_07153*-*SNOG_07156* were predicted within a ∼7.6 kb region containing *SnTox1*. Primers were designed (Table S4) to amplify the gene region from start to stop codon for the four genes identified in the avirulent isolate Sn79-1087. Since all the genes except *SNOG_07154* were present in Sn79-1087, one new primer designed in *SNOG_07153* (20078g3R, Table S4) was used with the SNOG07155 forward primer to amplify the whole region in several virulent isolates as well as Sn79-1087. The amplified fragments with different sizes were cloned into the pCR-4 TOPO cloning vector (Invitrogen) for sequencing. The sequences from different isolates were analyzed manually to identify the variations in the *SnTox1* genomic region with the aid of the genome sequence (http://genome.jgi-psf.org/Stano1/Stano1.home.html).

### SnTox1 gene transformation into the avirulent isolate Sn79-1087

A ≈1.1 kb sequence of the *SnTox1* genomic region including a putative promoter and terminator was amplified from the Sn2000 isolate using primers 20078gF_*Xba*I and 20078gR_*Xba*I, each containing an *Xba*I restriction site sequence (Table S4). The amplified fragment was cloned into the pCR-4 TOPO vector (Invitrogen) for sequencing to verify the identity and *Xba*I restriction sites. The *SnTox1* gene fragment was then released from pCR-4 TOPO plasmid and cloned into the pDAN vector that carries the *cpc-1:hyg^R^* (hygromycin-resistance gene) cassette. The resulting plasmid, designated pDAN-*SnTox1* (Figure S4) containing the 1.1 kb genomic region containing SnTox1 and *hyg^R^* was used to transform Sn79-1087 protoplasts. Plasmid DNA was prepared through the regular alkaline lysis method as described by Sambrook and Russell [79] followed by the purification of the plasmid DNA using precipitation with PEG 8000 [79]. The plasmid DNA was linearized with *Eco*R V and concentrated to 1 µg/ µl for transformation. The fungal protoplasting and PEG-mediated transformation followed the procedure described by Liu et al. [14]. The regenerated clones were screened by PCR with primers 20078gF_XbaI and 20078gR_XbaI (Table S4) and verified by Southern analysis [76]. The culture filtrate production, and infiltration and fungal inoculation with Sn79-1087 and *SnTox1* transformed strains followed the protocol described previously [27], [45].

### *SnTox1* gene replacement in the virulent isolate Sn2000

The knock out of *SnTox1* was performed using a split marker strategy which employed two rounds of PCR to generate replacement fragments as described by Catlett et al. [80] (Figure S4). In the first round of PCR, the 800 bp of 5′ flanking region and 825 bp of 3′ flanking region of *SnTox1* were amplified from Sn2000 using two pairs of primers 20078KOF1 with 20078KOF2 and 20078KOF3 with 20078KOF4 (Figure S4, Table S4). Simultaneously, overlapping marker fragments *HY* and *YG* of the hygromycin phosphotransferase cassette (*HYG*) were amplified from pDAN with two pairs of primers, M13F with HY and M13R with YG (Table S4, Figure S4). All amplified fragments were gel purified and then used in a second round of PCR. Two reactions were set up for the second round of PCR with one to fuse and amplify the *SnTox1* 5′ flanking region with the HY fragment and the other to fuse and amplify the *SnTox1* 3′ flanking region with the YG fragment by adding the corresponding first round templates and primers. At least 100 µl of PCR reaction was set up for each reaction in the second round. Standard PCR conditions and Taq polymerase (NEB BioLabs) were used for both rounds of amplification except that round 2 used a longer extension time due to the longer template. A small amount of product from the second round of PCR was evaluated on a 1.0% agarose gel to ensure a successful fusion and amplification for each fragment. The remaining product was combined and concentrated by routine ethanol precipitation [79]. The pellet was finally re-suspended in 20 µl of TE (10 mM Tris and 1 mM EDTA) for transformation of Sn2000 protoplasts.

Fungal protoplasting and transformation followed the procedure described by Liu et al. [14]. The regenerated clones were screened using the PCR primers 20078KOF and 20078KOR (Table S4) which amplifies the partial coding region of *SnTox1* that was replaced by the hygromycin-resistance gene cassette. The ectopic transformant and two knock out transformants were verified by Southern blot analysis using the *SnTox1* coding region as a probe. Spores of the knock out and ectopic strains as well as wild type Sn2000 were inoculated onto wheat lines W-7984, and CS for testing the effect of the *SnTox1* knock out.

### QTL analysis of *SnTox1* knock out strains

The International Triticeae Mapping Initiative (ITMI) mapping population was originally used to map the *Snn1* gene, which confers sensitivity to SnTox1, and quantitative trait loci conferring resistance/susceptibility to Sn2000 [27], [45]. The same 106 recombinant inbred (RI) lines of this population were used to evaluate the genetically modified fungal strains including two Sn2000 SnTox1 knock out transformants (*Sn2000ΔSnTox1*–*9* and *Sn2000ΔSnTox1*–*15*), one Sn2000 ectopic transformant (*Sn2000ΔSnTox1-ECT*), and the wild type Sn2000 as a control. All strains were evaluated with three biological replications by inoculating their conidia onto the ITMI population as previously described [45]. The disease rating was conducted 7 days post inoculation using a 0–5 rating scale as described by Liu et al. [45]. Composite interval mapping with the average of three disease readings was performed as previously described [30].

### Disulfide bond prediction

The web-based program *DISULFIND* (http://disulfind.dsi.unifi.it/) was used to predict if a particular cysteine residue was involved in the formation of a disulfide bond (DB_state) as well as the confidence level of the prediction. The state of each cysteine residue was predicted as either involved (1) or not involved (0) in a DB. The scale of confidence of disulfide bonding state prediction ranges from 0 (low) to 9 (high) [46]. The web-based program *DiANNA 1.1* (http://clavius.bc.edu/~clotelab/DiANNA/) [47] was used to determine the best connectivity prediction of cysteine residues in SnTox1.

### qPCR analysis of *SnTox1* transcription during infection

The secondary leaves of CS (≈2 week old plants) were inoculated with a fungal strain modified from the avirulent isolate Sn79-1087 by addition of the *SnTox1* gene. The leaf tissues were collected from the inoculated leaves at 1 h, 3 h, 6 h, 12 h, 24 h, 2 d (day), 3 d, 4 d, 5 d, 6 d, and 7 d post inoculation. The RNA was extracted from leaf samples using the RNeasy Plant Mini Kit (QIAGEN) and treated with RNase-free DNase I (Promega). RNA sample quantification, cDNA synthesis, and gene transcript abundance analysis were performed as previously described [15]. The gene specific primers SnTox1qPCRF and SnTox1qPCRR (Table S4) designed within the two exons were used for the *SnTox1* gene in qPCR. The previously reported primers ActinF and ActinR [14] were used for the *S*. *nodorum* actin gene as internal control.

### Dithiothreitol (DTT) and heat treatment of the SnTox1 protein

Because all cysteine residues were predicted to form disulfide bonds, the protein stability of SnTox1 was investigated by DTT and heat treatment. For DTT treatment, the SnTox1 *P. pastoris* culture filtrate were treated with DTT (Fisher Scientific, Pittsburgh, PA) at final concentrations of 0, 20, or 40 mM and incubated at room temperature for 2 h or 4 h. For heat treatment, the *P. pastoris* culture filtrate was sealed in a 2 ml centrifuge tube and heated for 30 min or 1 h on a hot plate setting at 100°C. All treated culture filtrates were then infiltrated into CS leaves.

### DAB staining of hydrogen peroxide

The fully expanded secondary leaves of CS and CS ems237 were infiltrated with the culture filtrates from yeast transformed with *SnTox1* or culture filtrates from yeast transformed with an empty vector (as control). At 24, 48 and 72 hours post infiltration, leaf samples were collected and leaf segments with an infiltrated area were cut and stained in a freshly made 1 mg/ml 3′–3′ diaminobenzidine (DAB) (Sigma) solution. The preparation of a DAB staining solution and the staining process followed a procedure described by Thordal-Christensen et al. [48]. The stained leaf tissue was cleared for chlorophyll by placing them on a paper pre-soaked with ethanol/acetic acid solution (3∶1, V/V) in a petri dish and incubating overnight. The cleared leaves were rinsed and stored in a lactic acid/glycerol/H_2_O solution (1∶1∶1, v/v/v).

### DNA laddering analysis

The fully expanded secondary leaves of CS and CS ems237 were infiltrated with SnTox1 yeast culture filtrates and control culture filtrates. Leaf samples were taken at 1, 2, 4, 8, 10, 24, 36, 48, 60, and 72 hour post infiltration. The DNA was extracted from the collected leaf samples using the CTAB method [17]. The 5 µl of DNA from each sample were separated on a 2% agarose gel. The gel was stained in ethidium bromide solution for 1 hour, destained in water for 1 h and photographed using a Gel LOGIC 100 image system (Kodak).

### Wheat defense gene expression using RT-PCR and qPCR

The fully expanded secondary leaves of CS and CS ems237 were infiltrated with SnTox1 yeast culture filtrates and control culture filtrates. Five centimeter segments of infiltrated leaf tissue was collected at 1, 2, 4, 8, 10, 24, 36, 48, 60, and 72 hour post infiltration. Three leaves from different plants were collected as three replications for each time point. Total RNA was extracted from all leaf samples using the RNeasy plant kit (QIAGEN) and treated with RNase-free DNaseI (Promega). The RNA quantification and first strand cDNA synthesis were conducted as previously described [15]. Using the cDNA samples, we examined the expression of a total of 28 wheat genes that have been reported or predicted to be involved in the defense response [Lu et al. unpublished, 21] (Table S3). The RT-PCR and agarose gel electrophorsis were performed using a standard procedure. The same cDNA samples from the three replications were used to conduct the qPCR analysis for three genes: PR-1-A1, chitinase (PR-3), and thaumatin-like protein (PR-5) following the description by Faris et al. [15].

### Generation of GFP tagged strains and microscopy

The gGFP vector [81] was used to transform the green fluorescence protein gene into two fungal strains that were only different in the production of SnTox1. One was the avirulent Sn79-1087 that did not produce SnTox1 nor did it cause disease, and the other was an Sn79-1087 SnTox1 transformant (*Sn79+SnTox1A1*) that expressed SnTox1 and caused disease on *Snn1* lines. Since *Sn79+SnTox1A1* already carried the hygromycin resistance resulting from the SnTox1 transformation, the plasmid pII99 [82] containing geneticin resistance, was used with gGFP for co-transformation of this fungus. The plasmid DNA preparation, fungal protoplasting, and fungal transformation followed the same methods described above. For all transformations, at least 20 µg of each plasmid DNA linearized with the corresponding restriction enzyme (gGFP with *Bg*lII and pII99 with *Eco*RV) was used. The transformants with the strongest GFP signal were selected for both strains under the Nikon Eclipse TE-2000U microscope equipped with a GFP filter and UV light (Nikon, Japan).

The two GFP-tagged fungal strains were inoculated onto both genotypes of *Snn1* (CS) and *snn1* (CS ems237) as described in Liu et al. [45]. The inoculated leaves were collected at 1 h, 3 h, 6 h, 12 h, 24 h, 2 d, 4 d, and 7 d post inoculation. The leaves were cut into 5 cm long segments and directly mounted onto glass slides. The specimens were examined immediately using a Zeiss Axioplan 2 Imaging Research Microscope with ApoTome confocal component (Carl Zeiss Light Microscopy, Germany) equipped with filter blocks with spectral properties matching those of GFP.

### GenBank accession/ID numbers

The *S. nodorum* gene *SNOG_20078* has been deposited in Genbank with identity numbers of 5974395 for gene ID and XP_001797505.1 for protein ID. The nucleotide sequence of 12 different haplotypes of *SnTox1*, designated Tox1_H1–H13, was submitted to GenBank with accession numbers from JN791682 to JN791693. The other genes and proteins referred to in this paper included *Cladosporium fulvum* Avr4 protein (CAA69643.1), *Mycosphaerella fijiensis* Avr4-like protein (Protein ID: Mycfi1:87167), *Cercospora beticola* Avr4-like protein (GU574324), *Microsporum gypseum* Avr4-like protein (GeneID:10030079) and *Geomyces pannorum* Avr4-like protein (DY991214).

## Supporting Information

Figure S1 **SnTox1 nucleotide and deduced amino acid sequences.** The sequence of the 1.2 Kb *SnTox1* genomic region from SN15 is shown. The putative TATA box is highlighted in red, the 5′ and 3′ UTRs are highlighted in yellow and the start and stop codons are indicated in bold. The introns are highlighted in gray. The underlined sequences are the primers that were used to amplify the genomic region (∼1.1 kb) for transformation into the avirulent isolate Sn79-1087. The translated protein sequence is shown under the DNA sequence of the coding region with the first 17 amino acids highlighted in blue as the predicted signal peptide. The 16 cysteine residues are highlighted in green.
(TIF)

Figure S2 **Analysis of SnTox1 protein expressed in** ***Pichia pastoris*** **. A.** Western blot analysis of the SnTox1 protein expressed in *Pichia pastoris*. M: protein size marker (BIO-RAD, Cat#161-0377). 1 and 2: Independently prepared protein samples from culture filtrates of *P*. *pastoris* transformed with *SNOG_20078*; 3 and 4: Independently prepared protein samples from culture filtrates of *P*. *pastors* transformed with an empty vector. **B.** SDS-PAGE with Coomassie blue 250 staining to visualize the total protein in each sample. The same amount of each protein sample shown in panel A were loaded onto the gel.
(TIF)

Figure S3 **Amino acid sequence alignment of SnTox1 with its homologs obtained from BlastP searches.** The partial protein sequence of SnTox1 was aligned with three homologs, SNOG06478 from *Stagonospora nodorum*, and PTRT04748 and PTRT03544 from *Pyrenophora tritici-repentis.*
(TIF)

Figure S4 **Molecular manipulation and characterization in** ***SnTox1*** **gene transformation and disruption. A**. Map of the pDAN vector containing the *SnTox1* genomic region that was used to transform the avirulent isolate Sn79-1087. The plasmid contained the Hygromycin resistance gene (*Hyg^R^*) as a selectable marker. To facilitate cloning, the *Xba*I restriction site was incorporated into the primers that were used to amplify the *SnTox1* genomic region. The plasmid was linearized with *Eco*RV before transformation. **B**. Southern blot analysis of fungal transformants for *SnTox1* integration. The *SnTox1* genomic region (1.1 kb) was amplified from the virulent isolate Sn2000 (V) and transformed into the avirulent isolate Sn79-1087 (A). All transformants (No. 1–7) contained *SnTox1* insertion except No. 2 based on the Southern analysis using the *SnTox1* coding region as a probe. **C**. A PCR-based split marker strategy for replacement of the *SnTox1* gene in isolate Sn2000. Overlapping PCR was used to fuse the 5′ flanking region and 3′ flanking region with the HY and YG fragments which were amplified from the *Hyg^R^*. The two fused PCR fragments were used to transform Sn2000 to replace the SnTox1 coding region through homologous recombination. The green boxed region was amplified and used as a probe in Southern analysis of the SnTox1 disruption. **D**. Southern blot analysis of fungal transformants for *SnTox1* knock out. Genomic DNA from the wild type, knock out, and ectopic fungal strain was digested with *Xba*I and blotted to a nylon membrane, which was then hybridized with a probe from the deleted region of *SnTox1* (green box in C). The wild type (lane 1) and ectopic type, *Sn2000ΔSnTox1-ECT* (lane 4) contained a 5.0 kb fragment, but the fragment was absent in the two disrupted strains, *Sn2000ΔSnTox1-9* and *15* (lane 2 and 3).
(TIF)

Figure S5 **Gene expression patterns for** ***SnToxA*** **,** ***SnTox3*** **and** ***SnTox1*** **during infection revealed by microarray analysis.** The expression level of the three effector genes, *SnToxA*, *SnTox3* and *SnTox1*, was examined and compared to that of the *Act1* gene at 3, 5, 7 and 10 days after inoculation. The x axis shows the number of days post-infection. The y axis represents relative gene expression levels normalized to *Act1*.
(TIF)

Figure S6 **A close-up of a necrotic lesion induced by a GFP-tagged SnTox1 transformed fungal strain.** **A.** Microscopic examination under normal light; **B.** Microscopic examination under UV light with GFP filter indicating the extensive growth of the fungus within the lesion of dead cells (200 × magnification).
(TIF)

Table S1 ***SnTox1*** **distribution and haplotypes in a global collection of** ***S. nodorum*** **isolates.**
(DOC)

Table S2 ***S*** **.** ***nodorum*** **isolates and its related fungal species used in dot blot analysis of** ***SnTox1*** **presence.**
(DOC)

Table S3 **Defense response genes investigated in SnTox1-** ***Snn1*** **interaction.**
(DOC)

Table S4 **Primers used in this research.**
(DOC)
