# Supplementary figures and images for: The Cysteine Rich Necrotrophic Effector SnTox1 Produced by Stagonospora nodorum Triggers Susceptibility of Wheat Lines Harboring Snn1
Source: PLoS Pathog. 2012 Jan 5;8(1):e1002467. doi: 10.1371/journal.ppat.1002467 (PMC3252377; doi:10.1371/journal.ppat.1002467)

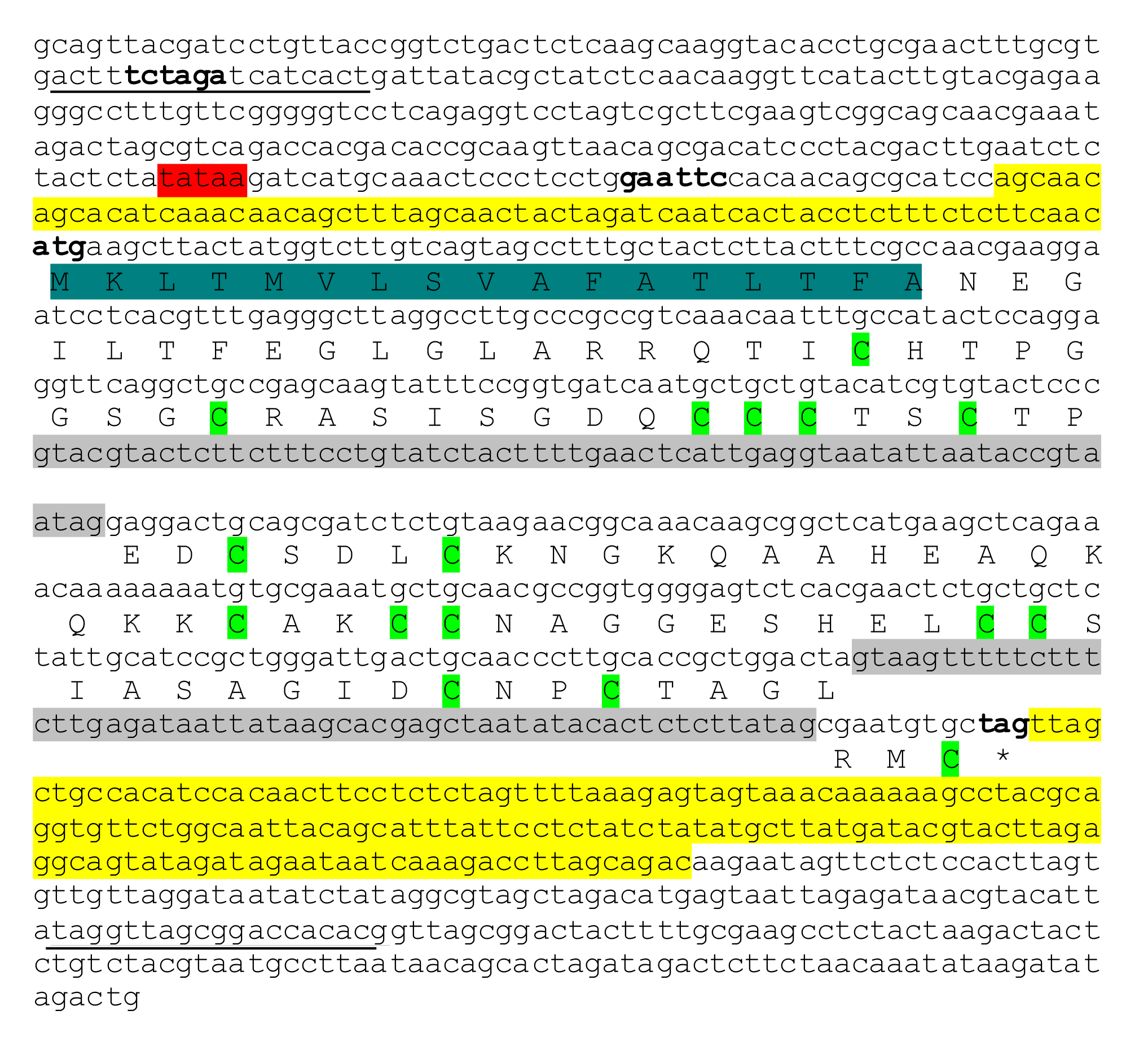

Supplement: Figure S1 — SnTox1 nucleotide and deduced amino acid sequences. The sequence of the 1.2 Kb SnTox1 genomic region from SN15 is shown. The putative TATA box is highlighted in red, the 5′ and 3′ UTRs are highlighted in yellow and the start and stop codons are indicated in bold. The introns are highlighted in gray. The underlined sequences are the primers that were used to amplify the genomic region (∼1.1 kb) for transformation into the avirulent isolate Sn79-1087. The translated protein sequence is shown under the DNA sequence of the coding region with the first 17 amino acids highlighted in blue as the predicted signal peptide. The 16 cysteine residues are highlighted in green. (TIF) [file ppat.1002467.s001.tif]

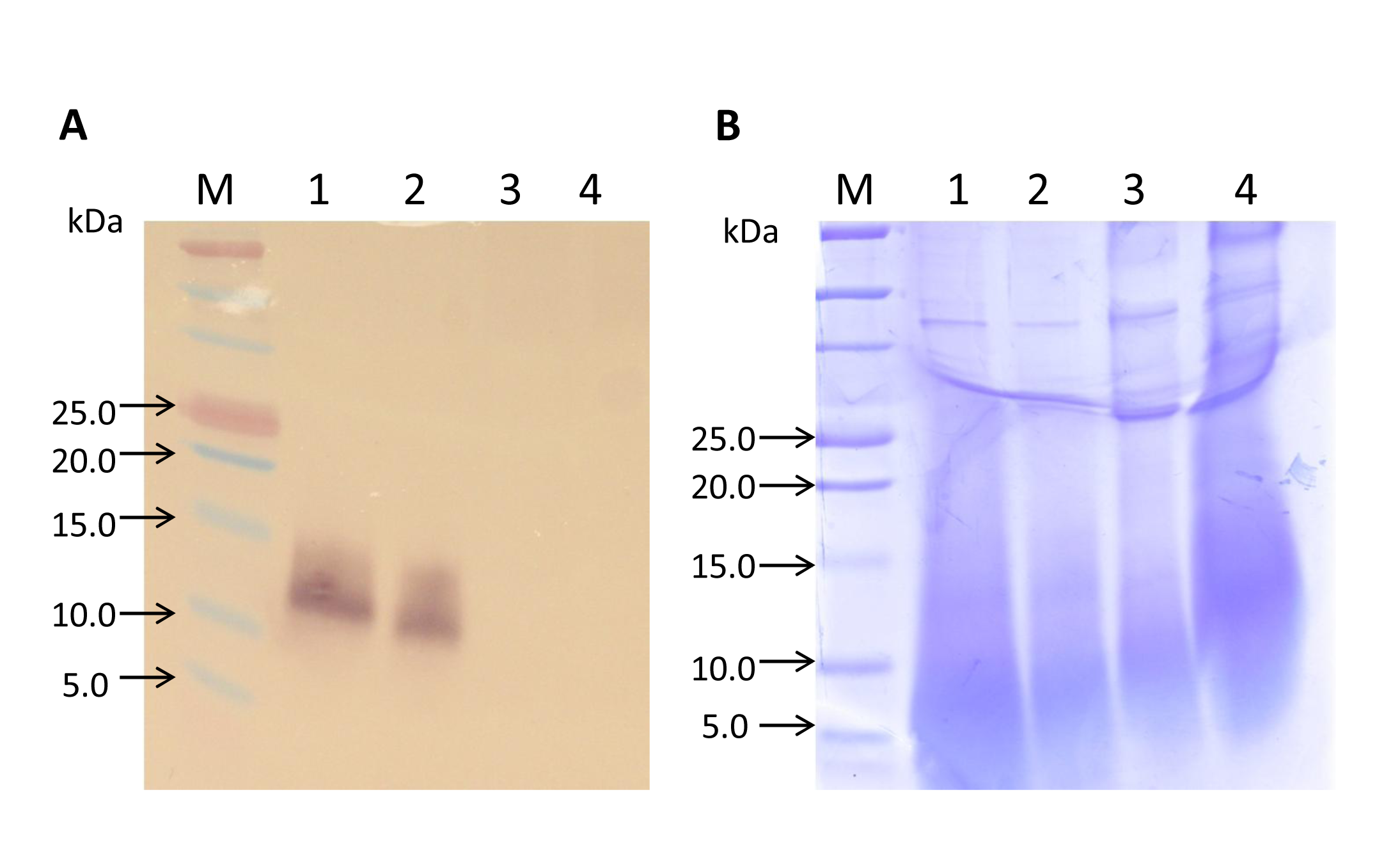

Supplement: Figure S2 — Analysis of SnTox1 protein expressed in Pichia pastoris . A. Western blot analysis of the SnTox1 protein expressed in Pichia pastoris. M: protein size marker (BIO-RAD, Cat#161-0377). 1 and 2: Independently prepared protein samples from culture filtrates of P. pastoris transformed with SNOG_20078; 3 and 4: Independently prepared protein samples from culture filtrates of P. pastors transformed with an empty vector. B. SDS-PAGE with Coomassie blue 250 staining to visualize the total protein in each sample. The same amount of each protein sample shown in panel A were loaded onto the gel. (TIF) [file ppat.1002467.s002.tif]

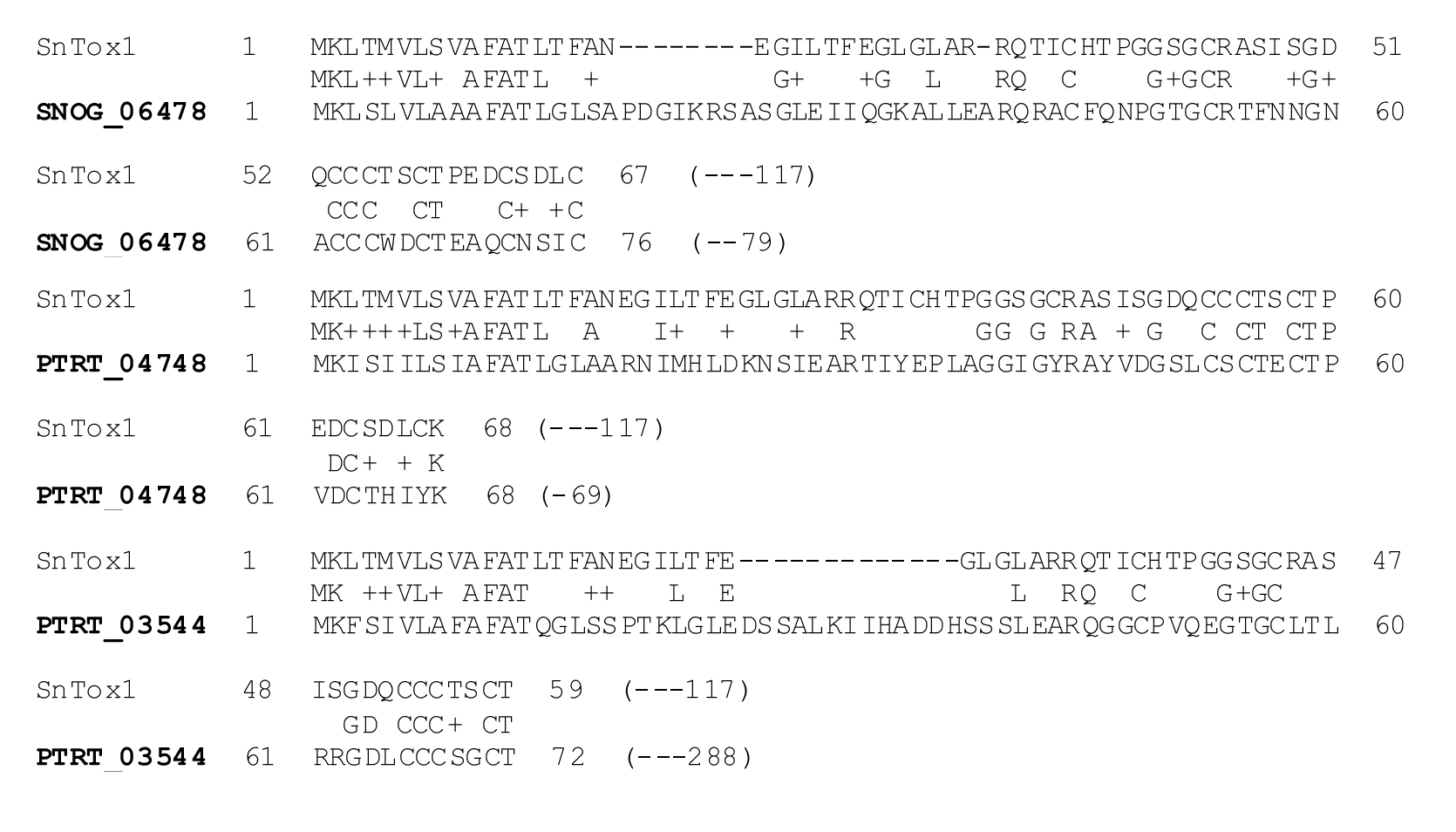

Supplement: Figure S3 — Amino acid sequence alignment of SnTox1 with its homologs obtained from BlastP searches. The partial protein sequence of SnTox1 was aligned with three homologs, SNOG06478 from Stagonospora nodorum, and PTRT04748 and PTRT03544 from Pyrenophora tritici-repentis. (TIF) [file ppat.1002467.s003.tif]

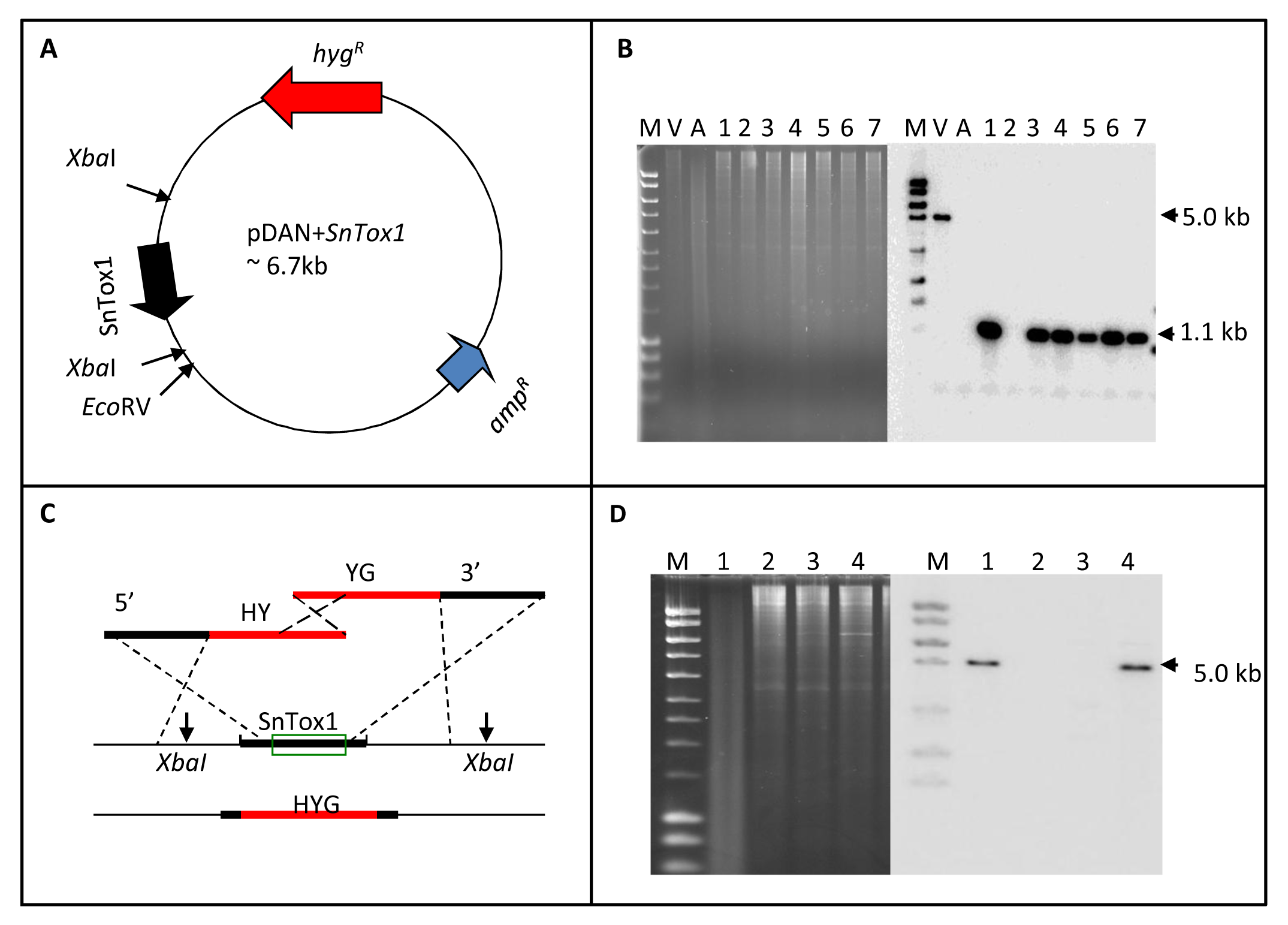

Supplement: Figure S4 — Molecular manipulation and characterization in SnTox1 gene transformation and disruption. A. Map of the pDAN vector containing the SnTox1 genomic region that was used to transform the avirulent isolate Sn79-1087. The plasmid contained the Hygromycin resistance gene (HygR) as a selectable marker. To facilitate cloning, the XbaI restriction site was incorporated into the primers that were used to amplify the SnTox1 genomic region. The plasmid was linearized with EcoRV before transformation. B. Southern blot analysis of fungal transformants for SnTox1 integration. The SnTox1 genomic region (1.1 kb) was amplified from the virulent isolate Sn2000 (V) and transformed into the avirulent isolate Sn79-1087 (A). All transformants (No. 1–7) contained SnTox1 insertion except No. 2 based on the Southern analysis using the SnTox1 coding region as a probe. C. A PCR-based split marker strategy for replacement of the SnTox1 gene in isolate Sn2000. Overlapping PCR was used to fuse the 5′ flanking region and 3′ flanking region with the HY and YG fragments which were amplified from the HygR. The two fused PCR fragments were used to transform Sn2000 to replace the SnTox1 coding region through homologous recombination. The green boxed region was amplified and used as a probe in Southern analysis of the SnTox1 disruption. D. Southern blot analysis of fungal transformants for SnTox1 knock out. Genomic DNA from the wild type, knock out, and ectopic fungal strain was digested with XbaI and blotted to a nylon membrane, which was then hybridized with a probe from the deleted region of SnTox1 (green box in C). The wild type (lane 1) and ectopic type, Sn2000ΔSnTox1-ECT (lane 4) contained a 5.0 kb fragment, but the fragment was absent in the two disrupted strains, Sn2000ΔSnTox1-9 and 15 (lane 2 and 3). (TIF) [file ppat.1002467.s004.tif]

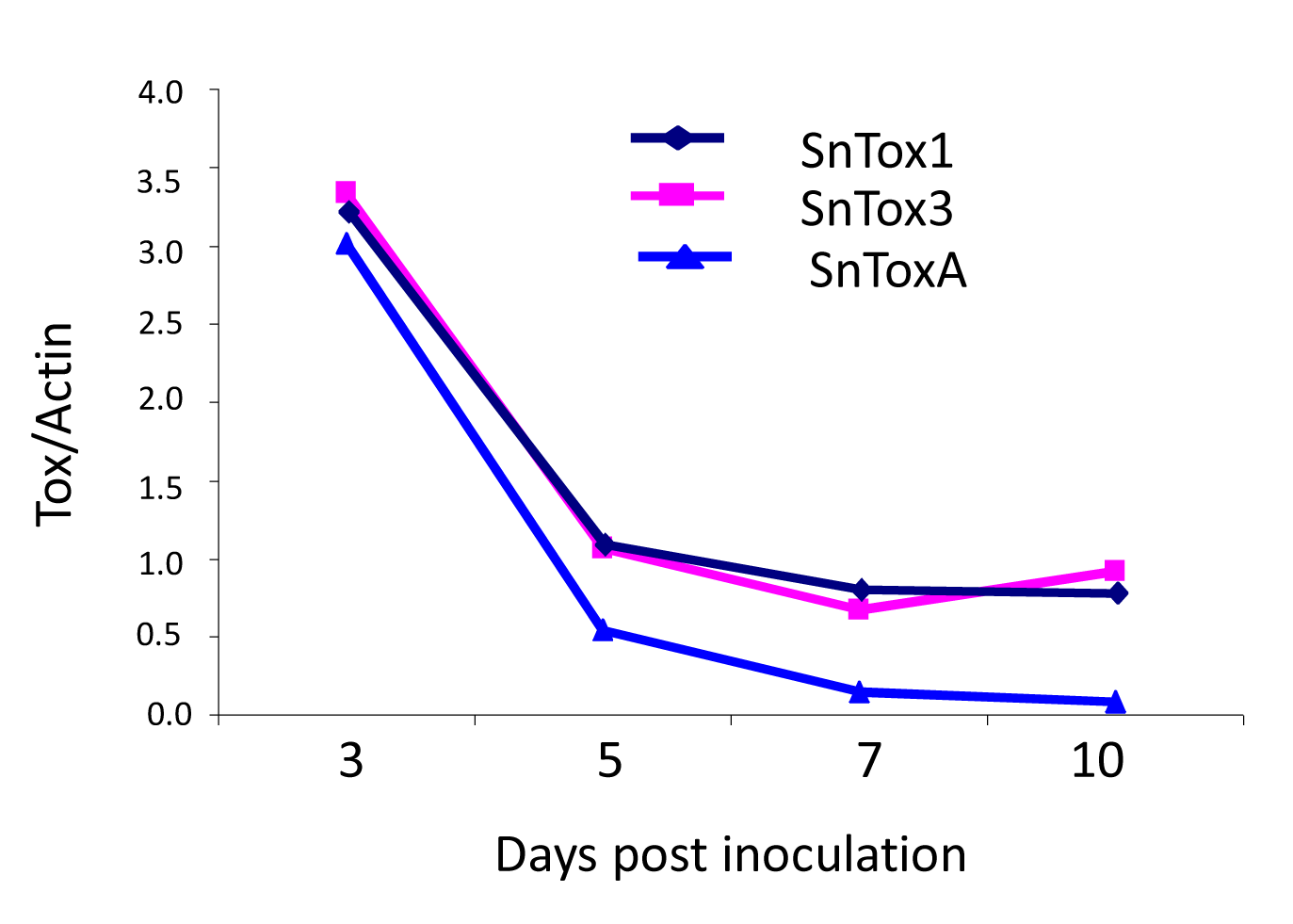

Supplement: Figure S5 — Gene expression patterns for SnToxA , SnTox3 and SnTox1 during infection revealed by microarray analysis. The expression level of the three effector genes, SnToxA, SnTox3 and SnTox1, was examined and compared to that of the Act1 gene at 3, 5, 7 and 10 days after inoculation. The x axis shows the number of days post-infection. The y axis represents relative gene expression levels normalized to Act1. (TIF) [file ppat.1002467.s005.tif]

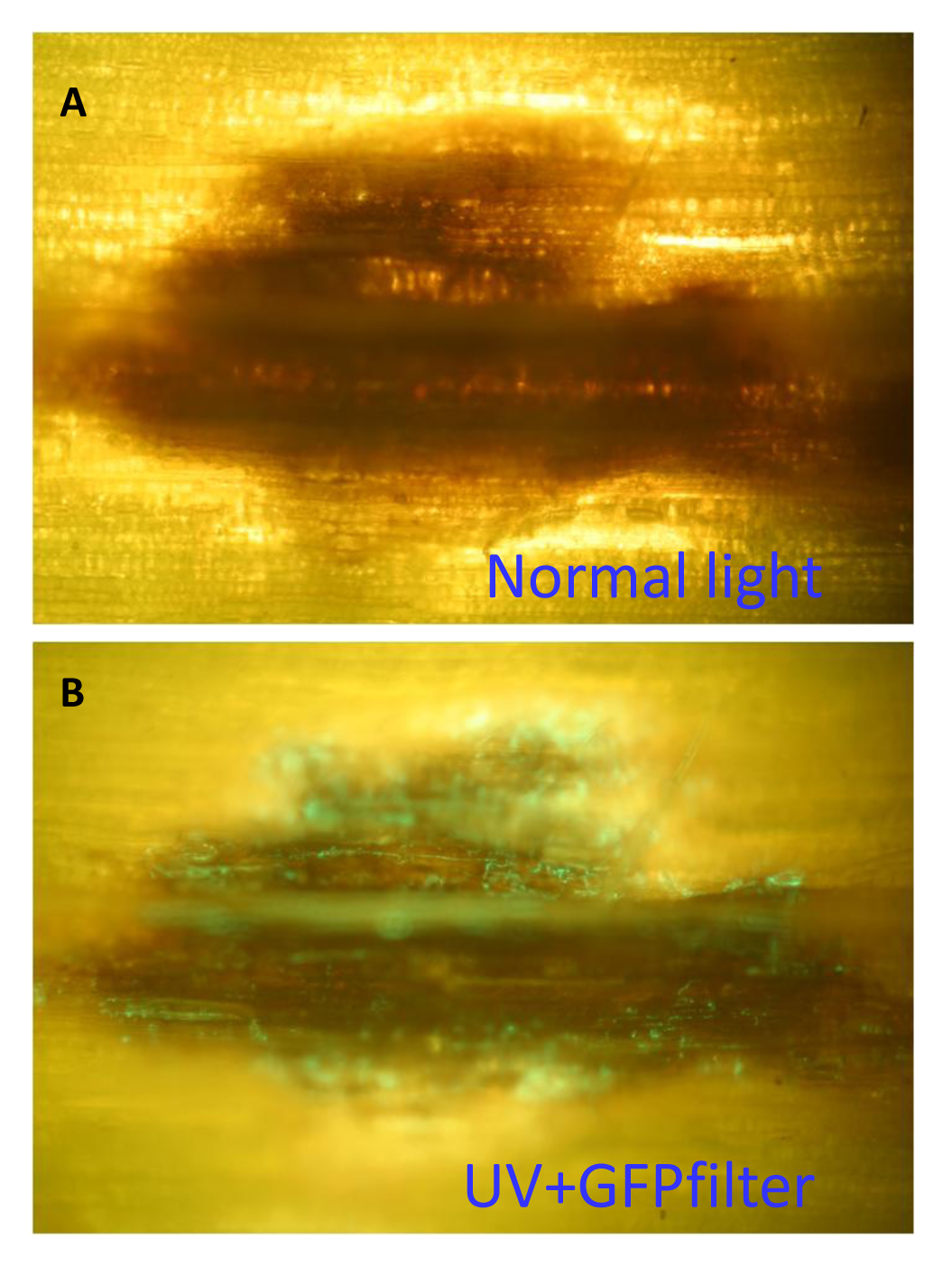

Supplement: Figure S6 — A close-up of a necrotic lesion induced by a GFP-tagged SnTox1 transformed fungal strain. A. Microscopic examination under normal light; B. Microscopic examination under UV light with GFP filter indicating the extensive growth of the fungus within the lesion of dead cells (200 × magnification). (TIF) [file ppat.1002467.s006.tif]
