# Supplementary material for: The Cysteine Rich Necrotrophic Effector SnTox1 Produced by Stagonospora nodorum Triggers Susceptibility of Wheat Lines Harboring Snn1
Source: PLoS Pathog. 2012 Jan 5;8(1):e1002467. doi: 10.1371/journal.ppat.1002467 (PMC3252377; doi:10.1371/journal.ppat.1002467)
Supplement: Table S1 — SnTox1 distribution and haplotypes in a global collection of S. nodorum isolates. (DOC) [file ppat.1002467.s007.doc]

Table S1. *SnTox1* distribution and haplotypes in a global collection of *S. nodorum* isolates.

| Population | Location | N* | *SnTox1+^†^* | Year | Collector |
| --- | --- | --- | --- | --- | --- |
| Australia |  | 73 | 71 | 2001 | B. McDonald |
| Central Asia | Kazakhstan | 31 | 21 | 2003,2004 | H. Maraite, E. Duvellier |
|  | Russia | 9 | 6 | 2003 | H. Maraite, E. Duvellier |
|  | Tajikistan | 7 | 5 | 2004 | H. Maraite, E. Duvellier |
|  | Kirgizstan | 1 | 1 | 2003 | H. Maraite, E. Duvellier |
| East Asia | China | 102 | 84 | 2001 | R. Wu |
| Europe | Denmark | 55 | 33 | 2005 | E. Stukenbrock, H. J. L. Jogensen |
|  | Sweden | 54 | 49 | 2005 | E. Blixt |
|  | Switzerland | 176 | 174 | 1994 | S. Keller |
| Middle East | Iran | 33 | 30 | 2005 | R. Sommerhalder |
| North America | New York | 46 | 35 | 1991 | G. Bergstrom |
|  | North Dakota | 47 | 45 | 1993 | L. Francl |
|  | North Carolina | 24 | 6 | 2005 | C. Cowger |
|  | Texas | 6 | 4 | unknown | B. McDonald |
|  | Ohio | 13 | 10 | unknown | P. Lipps |
| South America | Brazil | 3 | 2 | unknown | F. Santana |
| South Africa |  | 97 | 85 | 1995, 2007 | P. Crous, Z. Pretorius |
| Global |  | 777 | 661 | NA‡ | NA‡ |

*Number of isolates studied

†Number of isolates containing *SnTox1* gene.

‡ Not applicable
