# Supplementary material for: The Cysteine Rich Necrotrophic Effector SnTox1 Produced by Stagonospora nodorum Triggers Susceptibility of Wheat Lines Harboring Snn1
Source: PLoS Pathog. 2012 Jan 5;8(1):e1002467. doi: 10.1371/journal.ppat.1002467 (PMC3252377; doi:10.1371/journal.ppat.1002467)
Supplement: Table S2 — S . nodorum isolates and its related fungal species used in dot blot analysis of SnTox1 presence. (DOC) [file ppat.1002467.s008.doc]

Table S2. *Stagonospora nodorum* isolates and its related fungal species used in dot blot analysis of *SnTox1* presence.

| ID* | *Fungal species* | Geographic origin | Grid | Dot blot† |
| --- | --- | --- | --- | --- |
| SNChi01 40a | *S. nodorum* | China | A1 | Y |
| SNChi01 41a | *S. nodorum* | China | A2 | Y |
| SNChi01 45a | *S. nodorum* | China | A3 | Y |
| SNChi01 51a | *S. nodorum* | China | A4 | Y |
| SNChi01 52a | *S. nodorum* | China | A5 | Y |
| SNChi01 49B | *S. nodorum* | China | A6 | Y |
| SNChi01 50B | *S. nodorum* | China | A7 | Y |
| SN99CH 1A3a | *S. nodorum* | Switzerland | A8 | Y |
| SN99CH 1A4a | *S. nodorum* | Switzerland | A9 | Y |
| SN99CH 1A5a | *S. nodorum* | Switzerland | A10 | Y |
| SN99CH 1A7a | *S. nodorum* | Switzerland | A11 | Y |
| SN99CH 1A8a | *S. nodorum* | Switzerland | A12 | Y |
| SN99CH 1A9a | *S. nodorum* | Switzerland | B1 | Y |
| SN99CH 1A1b | *S. nodorum* | Switzerland | B2 | Y |
| SNOV92X D4.1 | *S. nodorum* | TX, USA | B3 | N |
| SNOV92X F1.1 | *S. nodorum* | TX, USA | B4 | Y |
| SNOV92X F2.1 | *S. nodorum* | TX, USA | B5 | Y |
| SNOV92X H1.4 | *S. nodorum* | TX, USA | B6 | Y |
| SNOV92X H2.3 | *S. nodorum* | TX, USA | B7 | Y |
| SNOV92X D1.3 | *S. nodorum* | TX, USA | B8 | N |
| SNSA95.35 | *S. nodorum* | South Africa | B9 | Y |
| SNSA95.58 | *S. nodorum* | South Africa | B10 | Y |
| SNSA95.61 | *S. nodorum* | South Africa | B11 | Y |
| SNSA95.62 | *S. nodorum* | South Africa | B12 | Y |
| SNSA95.86 | *S. nodorum* | South Africa | C1 | Y |
| SNSA95.109 | *S. nodorum* | South Africa | C2 | Y |
| SNAus01 1A1 | *S. nodorum* | Australia | C3 | Y |
| SNAus01 1A2 | *S. nodorum* | Australia | C4 | Y |
| SNAus01 1B2 | *S. nodorum* | Australia | C5 | Y |
| SNAus01 1C1 | *S. nodorum* | Australia | C6 | Y |
| SNAus01 1C9 | *S. nodorum* | Australia | C7 | Y |
| SNAus01 1C10 | *S. nodorum* | Australia | C8 | Y |
| SNAus01 1D1 | *S. nodorum* | Australia | C9 | Y |
| SNAus01 1D2 | *S. nodorum* | Australia | C10 | Y |
| BrSn9870 | *S. nodorum* | Brazil | C11 | Y |
| BrSn9990 | *S. nodorum* | Brazil | C12 | Y |
| BrSn9991 | *S. nodorum* | Brazil | D1 | N |
| SnCp2052 | *S. nodorum* | Denmark | D2 | Y |
| AuSn15 | *S. nodorum* | Australia | D3 | Y |
| 79-1021 | *S. nodorum* | ND, USA | D4 | N |
| 79-1087 | *S. nodorum* | ND, USA | D5 | N |
| 82-4841 | *S. nodorum* | ND, USA | D6 | N |
| 82-5036 | *S. nodorum* | ND, USA | D7 | N |
| 82-5154-1 | *S. nodorum* | ND, USA | D8 | N |
| 83-6011-2 | *S. nodorum* | ND, USA | D9 | N |
| 84-6377-1 | *S. nodorum* | ND, USA | D10 | N |
| 84-6369-1 | *S. nodorum* | ND, USA | D11 | N |
| 85-7600 | *S. nodorum* | ND, USA | D12 | N |
| 85-7706 | *S. nodorum* | ND, USA | E1 | N |
| 98-13025 | *S. nodorum* | ND, USA | E2 | Y |
| 98-13082 | *S. nodorum* | ND, USA | E3 | Y |
| 98-13066 | *S. nodorum* | ND, USA | E4 | Y |
| 98-13115-2 | *S. nodorum* | ND, USA | E5 | Y |
| 98-13063-1 | *S. nodorum* | ND, USA | E6 | Y |
| 98-13042-1 | *S. nodorum* | ND, USA | E7 | Y |
| 98-13050-1 | *S. nodorum* | ND, USA | E8 | Y |
| 98-13091 | *S. nodorum* | ND, USA | E9 | Y |
| OHSn14 | *S. nodorum* | OH, USA | E10 | Y |
| OHSn65 | *S. nodorum* | OH, USA | E11 | Y |
| OHSn94 | *S. nodorum* | OH, USA | E12 | Y |
| OHSn97 | *S. nodorum* | OH, USA | F1 | Y |
| OHSn123 | *S. nodorum* | OH, USA | F2 | N |
| OHSn601 | *S. nodorum* | OH, USA | F3 | Y |
| OHSn850 | *S. nodorum* | OH, USA | F4 | Y |
| OHSn901 | *S. nodorum* | OH, USA | F5 | N |
| OHSn1102 | *S. nodorum* | OH, USA | F6 | N |
| OHSn1180 | *S. nodorum* | OH, USA | F7 | Y |
| OHSn1354 | *S. nodorum* | OH, USA | F8 | Y |
| OHsn1501 | *S. nodorum* | OH, USA | F9 | Y |
| OHSn1553 | *S. nodorum* | OH, USA | F10 | Y |
| SN2K | *S. nodorum* | ND, USA | F11 | Y |
| Sn50 | *S. nodorum* | ND, USA | F12 | Y |
| Sn69-1 | *S. nodorum* | ND, USA | G1 | Y |
| KXE02 Sn-1 | *S. nodorum* | ND, USA | G2 | Y |
| LDN02 Sn-2 | *S. nodorum* | ND, USA | G3 | Y |
| LDN02 Sn-3 | *S. nodorum* | ND, USA | G4 | Y |
| BBC03 Sn-5 | *S. nodorum* | ND, USA | G5 | Y |
| LDN03 Sn-4 | *S. nodorum* | ND, USA | G6 | Y |
| LDN03 Sn-5 | *S. nodorum* | ND, USA | G7 | Y |
| LDN03 Sn-6 | *S. nodorum* | ND, USA | G8 | Y |
| LDN03 Sn-10 | *S. nodorum* | ND, USA | G9 | Y |
| LDN03 Sn-11 | *S. nodorum* | ND, USA | G10 | Y |
| BBC04 Sn-4 | *S. nodorum* | ND, USA | G11 | Y |
| CARW04 Sn-2 | *S. nodorum* | ND, USA | G12 | Y |
| FG05 Sn-1 | *S. nodorum* | ND, USA | H1 | Y |
| LDN05 Sn-4 | *S. nodorum* | ND, USA | H2 | Y |
| LDN06 Sn-1 | *S. nodorum* | ND, USA | H3 | Y |
| LDN07 Sn-3 | *S. nodorum* | ND, USA | H4 | Y |
| ND89-19 | *P. teres f. teres* | ND, USA | H5 | N |
| Pti2 | *P. tritici-repentis* | ND, USA | H6 | N |
| Sm18A | *P. bromi* | MN, USA | H7 | N |
| S. tr. 9715 | *M. graminicola* | **?** | H8 | N |

*Isolates in red are avirulent to wheat.

†Y=*SnTox1* present and N=*SnTox1* absent. Of 79 virulent isolates, only six do not contain *SnTox1*.
