# Supplementary material for: The Cysteine Rich Necrotrophic Effector SnTox1 Produced by Stagonospora nodorum Triggers Susceptibility of Wheat Lines Harboring Snn1
Source: PLoS Pathog. 2012 Jan 5;8(1):e1002467. doi: 10.1371/journal.ppat.1002467 (PMC3252377; doi:10.1371/journal.ppat.1002467)
Supplement: Table S3 — Defense response genes investigated in SnTox1- Snn1 interaction. (DOC) [file ppat.1002467.s009.doc]

Table S3. Defense response genes investigated in SnTox1-*Snn1* interaction

| Target gene | Primer | Sequence | Size (bp) | Reference | Regulated by SnTox1* |
| --- | --- | --- | --- | --- | --- |
| *PR-1-A1(unknown function)* | PR1A1-atgF | ATGGAGACGCCCAAGCTGGCCATTT | 495 | this study | Yes |
|  | PR1A1-taaR | TTAGTATGGTTTCTGTCCAACAAC |  |  |  |
| *PR1-A2(unknown function)* | PR1A2-atgF | ATGCAGACGCCCAAGCTAGCCATCT | 495 | this study | No |
|  | PR1A2-tagR | CTACTAGTATGGTTTCTGTCCAATG |  |  |  |
| *PR2 (Glucanase )* | PR2-atgF | ATGGCTGGAAAGGATGTTGC | 1005 | this study | No |
|  | PR2-taaR | TTAGAACTGGATGTTGTAGGCC |  |  |  |
| *PR3 (Chitinase)* | PR3-atgF | ATGAGAGCGTTCGCGCTGTTT | 957 | this study | No |
|  | PR3-taaR | TTAAGCGAAGGGCCTCTGATT |  |  |  |
| *PR4-1 (Chitin binding protein)* | PR4a-atgF | ATGGCCGCACGCCCGATGCT | 441 | this study | Yes/No |
|  | PR4a-tagR | CTAGTCGCGGCAGTCGACGA |  |  |  |
| *PR4-2 (Chitin binding protein)* | PR4b-atgF | ATGGCCGGACGCATGGCGCTT | 438 | this study | Yes/no |
|  | PR4b-tagR | TCAGTTGTCGCCGCAGTCGA |  |  |  |
| *PR5 (Thaumatin)* | PR5-atgF | ATGGCAACGGTGACCACCGG | 750 | this study | No |
|  | PR5-tagR | CTAGCGGCGGTGGGGGCAGA |  |  |  |
| *Caspase* | CASc-237F | TGCAGCTACGGCGACTCCCTGGT | 633 | this study | No |
|  | CASc-tagR | CTACATGCAGAACGGCTCGCGCT |  |  |  |
| *CNGC4 (cyclic nucleotide-gated ion channel 4)* | TaCNGC4-F1 | GATGGCGCTCGACTTCTTCGTCAT | 1156 | this study | No |
|  | TaCNGC4-R1 | CAGCAGCTCGTCGCCCGTGAAGTT |  |  |  |
| *RbohB (NADPH oxidase)* | TaRbohB-120F | CAGCACTCATCATGGAAGAGCTT | 352 | this study | No |
|  | TaRbohB-471R | CCCTTTGCAGTGGCCACACAGTA |  |  |  |
| *RbohC(NADPH oxidase)* | TaRbohC-217F | TCACTGACTGCAACGCAAGAT | 369 | this study | No |
|  | TaRbohC-583R | CTTGATACACACTCGAGCAAT |  |  |  |
| *RbohD (NADPH oxidase)* | TaRbohD-1289F | TCGTCTACGCGCTGCTCATCGT | 532 | this study | No |
|  | TaRbohD-1820R | ACGATGTCGTATTGCTTGTAGT |  |  |  |
| *SOD1(Superoxide dismutase)* | TaSOD1-252F | CCGCTCAGAGCCTCCTCTTT | 354 | this study | Yes/NO |
|  | TaSOD1-605R | CACCATGTGTCAGGCCGTTT |  |  |  |
| *PER22 (Peroxidase)* | TaPER22-180F | CCCGGACGCGTACAAGATCGT | 376 | this study | No |
|  | TaPER22-555R | TTTCCCGGCTTCGAAGAAGTT |  |  |  |
| *PAL1 (Phenylalanine ammonia-lyase)* | TaPAL1-577F | TCGAGGCCATCACCAAGCTCAT | 355 | this study | Yes/no |
|  | TaPAL1-931R | TTGCCGTTCATCACCTCGCAGA |  |  |  |
| *CHS1 (Chalcone synthase 1)* | TaCHS1-339F | GCTCACCTTCCACCTGCTCAA | 263 | this study | yes/no |
|  | TaCHS1-600R | GCATCTCATCCATGATGAAGA |  |  |  |
| TaMPK3 (mitogen-activated protein kinase) | TaMPK3F | TACATGAGGCACCTGCCGCAGT | nd† | Rudd et al., 2008 | No |
|  | TaMPK3R | GGTTCAACTCCAGGGCTTCGTTG |  |  |  |
| TaMPK 6(mitogen-activated protein kinase) | TaMPK6F | GAAGATATATCCGCCAACTTCCCCG | nd† | Rudd et al., 2008 | No |
|  | TaMPK6R | CGCATGCTGCTCGAAGTCAAAGC |  |  |  |
| Allene oxide cyclase | MB36 | TTGAGCTTGACCTGGCCGTAGG | 244 | Pandelova et al., 2009 | No |
|  | MB37 | TTGAGCTTGACCTGGCCGTAGG |  |  |  |
| Allene oxide synthase | MB30 | ACGGCGAGGGAGTTCGTCGG | 190 | Pandelova et al., 2009 | No |
|  | MB31 | GAAGGTGTCGTACCGGGAGG |  |  |  |
| Brassionsteroid insensitive 1 | MB51 | ATGTCACCTGCAACAACGT | 195 | Pandelova et al., 2009 | No |
|  | MB52 | ATCAAGGCTGACCAGGCGTG |  |  |  |
| Chlorophyll a-b binding protein 2 | MB45 | GCCGCCTTGCCATGTTCTCCAT | 195 | Pandelova et al., 2009 | No |
|  | MB46 | ATATCAGACCACCCCGCCAGCTCTC |  |  |  |
| chitinase | MB25 | GCGGGCCCATCCAGATCTC | 255 | Pandelova et al., 2009 | yes |
|  | MB26 | CACACCGTACCCAGGCACCCT |  |  |  |
| Cinnamate-4-hydrolase | MB32 | ACGACTGAGAAGGGTGGCCA | 240 | Pandelova et al., 2009 | No |
|  | MB33 | CCTCTGTTCCTTGCGGATAACA |  |  |  |
| 4-Coumaryl-CoA ligase | MB34 | GGTTACCTGAACGACCCAGA | 148 | Pandelova et al., 2009 | No |
|  | MB35 | CCTGGAAGCCCTTGTACTTG |  |  |  |
| Gibberellin receptor GIDL2 | MB53 | AAGGGGAAGGCGGTCAAGGT | 208 | Pandelova et al., 2009 | No |
|  | MB54 | CGCTATAGGTATCACACAGG |  |  |  |
| Glutathione peroxidase | MB43 | CCTATTTTTCGCAAGGTCGACG | 182 | Pandelova et al., 2009 | No |
|  | MB44 | TCAATGCCGAGTGGGGACCA |  |  |  |
| Thaumatin | MB23 | TGGGCGCAGGCCAGACGTCC | 244 | Pandelova et al., 2009 | yes |
|  | MB24 | GGCAAGGTTGAAGCCGTCG |  |  |  |
| Phenylalanine ammonia-lyase | MB47 | CTTATTCGAACAATCGACCGCG | 180 | Pandelova et al., 2009 | yes/no |
|  | MB48 | CTCCTGCTCGAATTTGGCAA |  |  |  |
| Superoxide dismutase 1 | MB41 | TGTTACTGACTGCCATATCCCC | 180 | Pandelova et al., 2009 | yes/no |
|  | MB42 | GAAGATGACATCTAGCCCTG |  |  |  |
| 18S ribosomal RNA | 18S-F | GTGACGGGTGACGGAGAATT | 151 | Pandelova et al., 2009 |  |
|  | 18S-R | GACACTAATGCGCCCGGTAT |  |  |  |

*Yes = higher transcription in SnTox1-infiltrated leaves across all time points, NO= no or weak transcription, and yes/no= higher transcription in SnTox1-infiltrated leaves only at certain time points.

†nd= no data
