# Supplementary material for: The Cysteine Rich Necrotrophic Effector SnTox1 Produced by Stagonospora nodorum Triggers Susceptibility of Wheat Lines Harboring Snn1
Source: PLoS Pathog. 2012 Jan 5;8(1):e1002467. doi: 10.1371/journal.ppat.1002467 (PMC3252377; doi:10.1371/journal.ppat.1002467)
Supplement: Table S4 — Primers used in this research. (DOC) [file ppat.1002467.s010.doc]

Table S4. Primers used in this study.

| ID | Sequence | Tm  °C | Applications and notes |
| --- | --- | --- | --- |
| 20078CF_ECoRI | *GAATTC*ATGAAGCTTACTATGGTCTTGT | 60 | Amplifying SnTox1 coding region for yeast expression. Restriction site (italicized) were incorporated into primers. |
| 20078CR_ApaI | *GGGCCC*TGTGGCAGCTAACTAGCACA | 60 |
| 20078CF | ATGAAGCTTACTATGGTCTTGT | 60 | *SnTox1* specific primers for 5’ and 3’ RACE |
| 20078CR | TGTGGCAGCTAACTAGCACA | 60 |
| 20078KOF | GCCATACTCCAGGAGGTTCA | 60 | Amplifying SnTox1 region that was replace by *Hyg^R^* for selection of knockout transformants and Southern probe |
| 20078KOR | TTACTAGTCCAGCGGTGCAA | 60 |
| 20078g1F_XbaI | TACA*TCTAGA*CCTTCTTCCAT | 60 | Amplifying SnTox1 genomic region for transformation into Sn79-1087. Restriction site (italicized) were incorporated into primers. |
| 20078g1R_XbaI | AA*TCTAGA*CGTGTGGTCCGCTAACCTAT | 60 |
| 20078KOF1 | TTCGTGCGGACTTATCACAC | 60 | Disruption of *SnTox1* in Sn2000 isolates. The PCR-based split-marker strategy was used to replace most portion of SnTox1 with *Hyg^R^* . For fusion purpose, two primers were incorporated with reverse complementary sequence of M13F and M13R (italicized). |
| 20078KOF2 | *CACTGGCCGTCGTTTTACAACGTC*ACGTGAGGATTCCTTCGTTG | 60 |
| 20078KOF3 | *TCATGGTCATAGCTGTTTCCTGTG*TTAGCTGCCACATCCACAAC | 60 |
| 20078KOF4 | TCCAGCACCATAACTCCACA | 60 |
| M13F | GACGTTGTAAAACGACGGCCAGTG | 60 |
| M13R | CACAGGAAACAGCTATGACCATGA | 60 |
| HY | GGATGCCTCCGCTCGAAGTA | 60 |
| YG | CGTTGCAAGACCTGCCTGAA | 60 |
| SnTox1RT1F | CTCACGTTTGAGGGCTTAGG | 58 | qPCR analysis of *SnTox1* transcription during infection. |
| SnTox1RT1R | GGATGCAATAGAGCAGCAGA | 58 |
| ActinqPCR2f | CTGCTTTGAGATCCACAT | 58 | S. nodorum action gene as Internal control for *SnTox1* transcription analysis. |
| ActinqPCR2r | GTCACCACTTTCAACTCC | 58 |
| SNOG07153F | CTATGCCAAATCCTGAGTACTC | 60 | Investigation of *SnTox1* genomic region. The primers were designed for each individual genes (SNOG07153 to SNOG07155) surrounding SnTox1 and used to investigate their presence in virulent and avirulent isolates. The primer SNOG07154F and 20078g were used to amplify 7.6 kb region from both virulent and avirulent isolates. |
| SNOG07153R | AACGATGCCATAATACATCGCCAT | 60 |
| SNOG07154F | ATGGCGATGTATTATGGCATCGT | 60 |
| SNOG07154R | CTATGCCAAATCCTGAGTACTCAAC | 60 |
| SNOG07155F | ATGTGCAATTTCGCAGAAATCGT | 60 |
| SNOG07155R | TCAGCTCCCATCGACCCGCCCA | 60 |
| SNOG07156F | ATGTGTCAATTCGAGCTTCCCT | 60 |
| SNOG07156R | TAAGTCTGTTTCTTGAGAGT | 60 |
| 20078g3R | AATCGACGAGATGGATGGAG | 60 |
| Tox1Fcoding | TTACTATGGTCTTGTCAGTAGCC | 56 | Investigation of the presence of *SnTox1* in global S. nodorum isolates and haplotype variation of *SnTox1* in different isolates. |
| Tox1Rcoding | ACACTCTCTTATAGCGAATGTG | 56 |
| Tox1_XF | CATACTCCAGGAGGTTCAGG | 58 |
| Tox1_XR | GTCTCACGAACTCTGCTGC | 58 |
| Tox1UTR_F | AGA TCA TGC AAA CTC CCT CCT | 56 |  |
| Tox1UTR_R | TTC GCA AAA GTA GTC CGC TAA C | 56 |  |
| Tox1Fout | ATGATGCATAGAAGGGTACCAC | 56 |  |
| Tox1Rout | GCAGAAGGGAAAGCAACAA | 56 |  |
